# Supplementary material for: Efficient self-assembly of heterometallic triangular necklace with strong antibacterial activity
Source: Nat Commun. 2020 Jun 23;11:3178. doi: 10.1038/s41467-020-16940-z (PMC7311404; doi:10.1038/s41467-020-16940-z)
Supplement: Supplementary file 1 — Supplementary Information [file 41467_2020_16940_MOESM1_ESM.pdf]

Supplementary Information

Efficient Self-Assembly of Heterometallic Triangular Necklace with Strong Antibacterial Activity

Wu et al

Table of Contents

1. **Supplementary Methods**…………………….…..………….............................S2
2. **Supplementary Note 1:** Experimental details of DNA cleavage, bacterium-binding and antimicrobial tests ……………………………………………..…….…..….S9
3. **Supplementary Note 2:** Stability studies……………………………………..….S10
4. **Supplementary Tables**…………………………...…………………………S11
5. **Supplementary Figures**……………………………………………………….S12

1. Supplementary Methods

All solvents were dried according to standard procedures and all of them were degassed under N_2 for 30 minutes before use. All air-sensitive reactions were carried out under inert N_2 atmosphere. ^{1}H, ^{13}C NMR and ^{31}P NMR spectra were recorded on Bruker 500 MHz Spectrometer (^{1}H: 500 MHz; ^{31}P: 202 MHz; ^{13}C: 125 MHz) at 298 K. The ^{1}H and ^{13}C NMR chemical shifts are reported relative to the residual solvent signals. 2D DOSY were recorded on Bruker 500 MHz Spectrometer (^{1}H: 500 MHz) at 298 K. The ESI-TOF-MS spectra were acquired using an AccuTOF CS mass spectrometer (JMS-T100CS, JEOL, Tokyo, Japan). TEM images were recorded on a Tecnai G2 F30 (FEI Ltd.) and JEM-1011 (JEOL Ltd.).

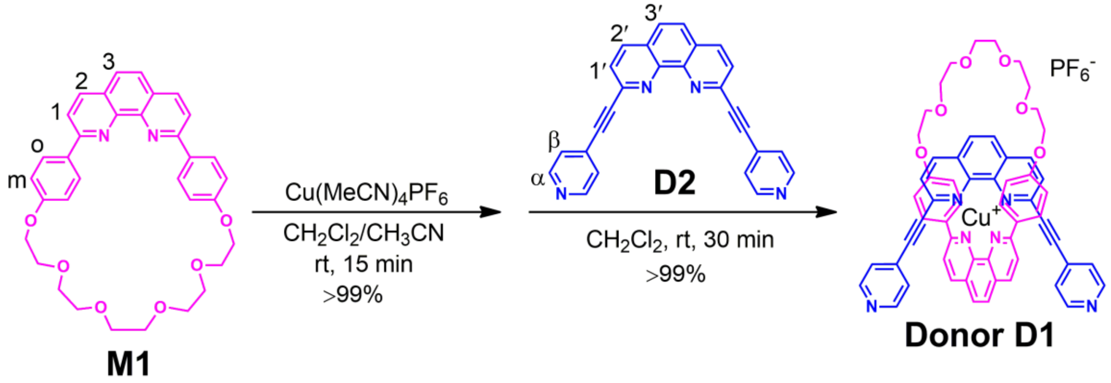

Supplementary Figure 1. The synthetic procedure for the pesudorotaxane-containing donor building block **D1**.

*Synthesis of donor **D1***. In a Schlenk flask, 1.0 equiv of macrocycle **M1** (23.91 mg, 0.042 mmol) was dissolved under argon in a 1:1 mixture of dichloromethane and acetonitrile. After addition of 1.0 equiv of $Cu(MeCN)_4PF_6$ (15.73 mg, 0.042 mmol), the reaction was stirred at room temperature under argon for 15 min. In a second Schlenk flask, 1.0 equiv of **D2** (16.06 mg, 0.042 mmol) was dissolved in dichloromethane and cannula-filtered into the first solution. The solution was stirred under argon at room temperature for an additional 30 min, followed by removal of the solvent in vacuo to dryness to afford quantitatively **D1** as a brown-red solid (yield = 48.61 mg, 99%). ^{1}H NMR (500 MHz, acetone-d_6) δ 8.85 (d, J = 10.0 Hz, 2H), 8.72 (d, J = 2.5 Hz, 2H), 8.45 (s, 2H), 8.33-8.10 (m, 8H), 8.03 (d, J = 10.0 Hz, 2H), 7.54 (d, J = 5.0 Hz, 4H), 6.41 (s, 4H), 6.05 (d, J = 10.0, 8.2 Hz, 4H), 3.86 (s, 4H), 3.75-3.73 (m, 4H), 3.61-3.59 (m, 4H), 3.51-3.49 (m, 4H), 3.46-3.44 (m, 4H). ESI-TOF-MS of **D1**: m/z calcd for $[C_{60}H_{48}N_6O_6CuPF_6]$ ($[M-PF_6^-]^+$): 1012.63, Found: 1011.2.

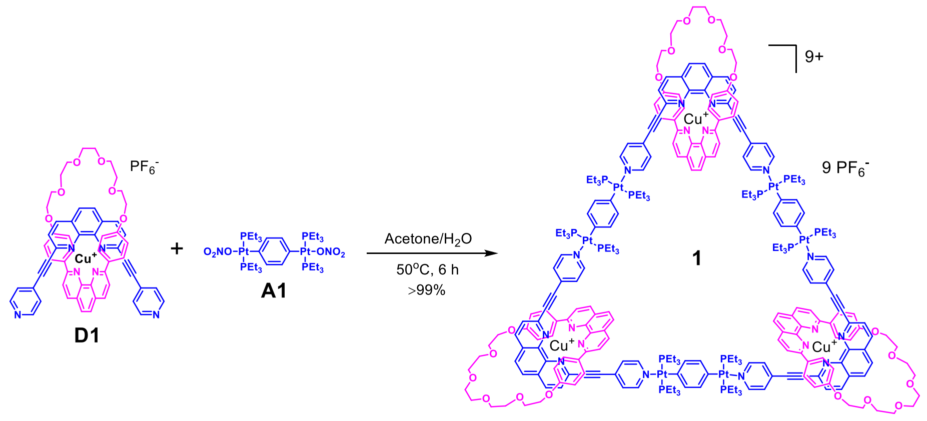

Supplementary Figure 2. The synthetic procedure for the molecular necklace **1**.

*Synthesis of molecular necklace **1**.* Self-assembly of molecular necklace **1** from donor **D1** and diplatinum acceptor **A1**. The dipyridyl donor ligand **D1** (8.87 mg, 7.66 μmol) and 180° organoplatinum acceptor **A1** (8.14 mg, 7.66 μmol) were weighed accurately into a glass vial. To the vial was added 2.0 mL acetone and 0.4 mL H_2O, and the reaction solution was stirred at room temperature for 6 hours. The molecular necklace **1** could also be obtained in one-pot reaction without purification of **D1**. Equal amount of **M1** (4.34 mg, 7.66 μmol) and $Cu(MeCN)_4PF_6$ (2.86 mg, 7.66 μmol) in dichloromethane and acetonitrile was stirred for 15 mins, and then same amount of **D2** (2.92 mg, 7.66 μmol) was added and was further stirred for 30 min. After removing the solvent under vacuum and redissolved in acetone/water, the 180° organoplatinum acceptor **A1** (8.14 mg, 7.66 μmol) was added and the above mixture was assembled in 6 h. The PF_6^- salt of **1** was synthesized by dissolving the NO_3^- salt of **1** in acetone/H_2O and adding a saturated aqueous solution of KPF_6 to precipitate the product, which was collected by vacuum filtration. Yield: 18.1 mg, 99%. ^{1}H NMR (500 MHz, acetone-d_6) δ 8.92 (d, J = 5.0 Hz, 4H), 8.74 (d, J = 5.0 Hz, 4H), 8.52 (s, 2H), 8.41 (s, 2 H), 8.35 (d, J = 10.0 Hz, 2H), 8.14 (d, J = 5.0 Hz, 2H), 7.57 (d, J = 5.0 Hz, 4H), 7.16 (s, 4H), 6.87 (d, J = 5.0 Hz, 4H), 6.10 (d, J = 5.0 Hz, 4H), 3.86 (s, 4H), 3.75 (d, J = 5.0 Hz, 4H), 3.63 (m, 4H), 3.53-3.49 (m, 8H), 1.46 (s, 24H), 1.21-1.05 (m, 36H). ^{13}C NMR (125 MHz, acetone-d_6) δ 159.4, 158.5, 152.3, 143.6, 142.3, 138.2, 137.9, 137.8, 136.6, 133.2, 131.4, 129.7, 128.9, 128.3, 128.2, 128.1, 126.5, 124.7, 113.2, 94.2, 88.1, 70.9, 70.6, 70.5, 68.66, 67.16, 12.5, 12.3, 12.2, 7.1. ^{31}P NMR (acetone-d_6, 202 MHz): δ 12.60 (s, $J_{Pt\text{-}P}$= 2715.3 Hz). ESI-MS: m/z: 1644.80 $[M-4PF_6]^{4+}$, 1287.21 $[M-5PF_6]^{5+}$.

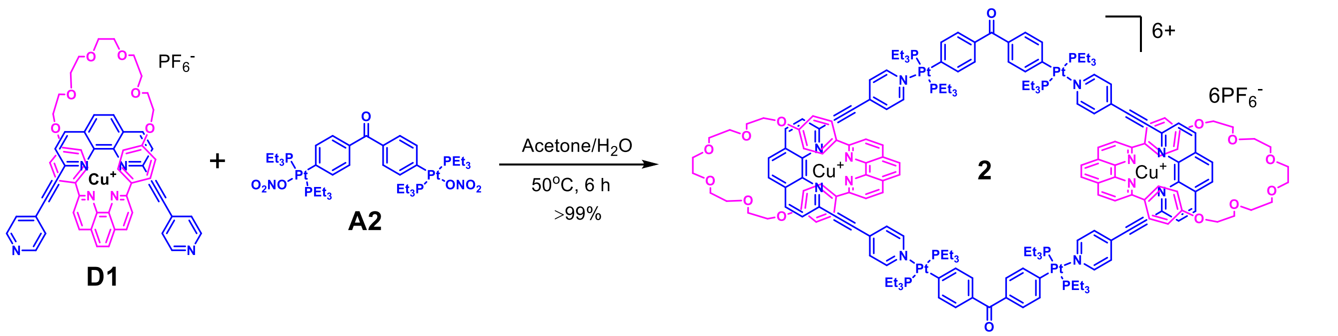

Supplementary Figure 3. The synthetic procedure for the [3]catenane **2**.

Synthesis of [3]catenane ***2****.* Self-assembly of **2** from donor **D1** and diplatinum acceptor **A2**. The dipyridyl donor ligand **D1** (7.30 mg, 6.31 μmol) and 120° organoplatinum acceptor **A2** (7.37 mg, 6.31 μmol) were weighed accurately into a glass vial. To the vial was added 2.0 mL acetone and 0.4 mL H_2O, and the reaction solution was stirred at room temperature for 6 hours. The PF_6^- salt of **2** was synthesized by dissolving the NO_3^- salt of **2** in acetone/H_2O and adding a saturated aqueous solution of KPF_6 to precipitate the product, which was collected by vacuum filtration. Yield: 15.72 mg, 99%. ^{1}H NMR (500 MHz, acetone-d_6) δ 8.96-8.92 (m, 4H), 8.76 (d, J = 5.0 Hz, 4H), 8.53 (s, 2H), 8.49 (s, 2H), 8.36 (d, J = 10.0 Hz, 2H), 8.16 (d, J = 5.0 Hz, 2H), 7.70 (d, J = 10.0 Hz, 4H), 7.60 (d, J = 10.0 Hz, 4H), 7.56 (d, J = 10.0 Hz, 4H), 6.83 (d, J = 5.0 Hz, 3H), 6.10 (d, J = 10.0 Hz, 4H), 3.87 (s, 4H), 3.77-3.75 (m, 4H), 3.64-3.62 (m, 4H), 3.54 (d, J = 5.0 Hz, 4H), 3.50 (d, J = 5.0 Hz, 4H), 1.47-1.46 (m, 24H), 1.24-1.18 (m, 36H). ^{13}C NMR (125 MHz, acetone-d_6) δ 196.3, 160.2, 159.5, 153.2, 144.4, 143.2, 138.9, 136.9, 134.0, 132.5, 130.8, 130.4, 130.2, 129.3, 129.0, 125.8, 114.0, 95.3, 88.8, 71.8, 71.4, 71.3, 69.5, 68.0, 13.3, 13.1, 13.0, 7.9. ^{31}P NMR (acetone-d_6, 202 MHz): δ 14.31 (s, $J_{Pt\text{-}P}$= 2642.4 Hz). ESI-MS: m/z: 1515.16 [M-3PF$_6$]$^{3+}$, 1100.33 [M-4PF$_6$]$^{4+}$, 851.23 [M-5PF$_6$]$^{5+}$.

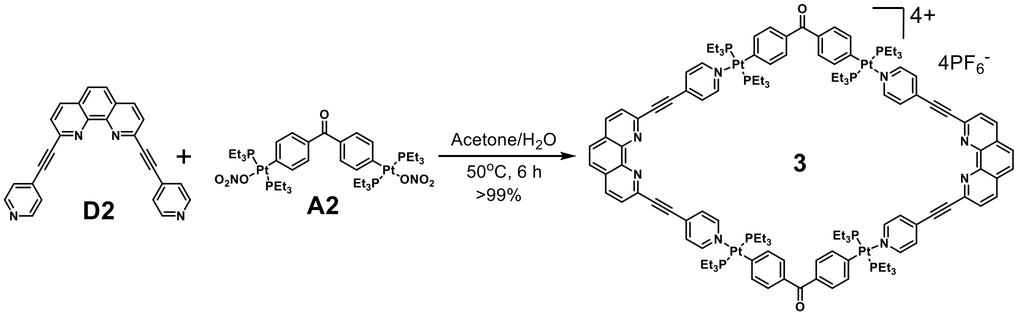

Supplementary Figure 4. The synthetic procedure for the metallacycle **3**.

*Synthesis of metallacycle **3***. Self-assembly of metallacycle **3** from donor **D2** and diplatinum acceptor **A2**. The dipyridyl donor ligand **D2** (4.36 mg, 11.4 μmol) and 120° organoplatinum acceptor **A2** (13.38 mg, 11.4 μmol) were weighed accurately into a glass vial. To the vial was added 2.0 mL acetone and 0.4 mL H_2O, and the reaction solution was stirred at room temperature for 6 hours. The PF_6^- salt of **3** was synthesized by dissolving the NO_3^- salt of **3** in acetone/H_2O and adding a saturated aqueous solution of KPF_6 to precipitate the product, which was collected by vacuum filtration. Yield: 19.36 mg, 99%. ^{1}H NMR (500 MHz, acetone-d_6) δ 9.16 (d, J = 5.0 Hz, 4H), 8.68 (d, J = 5.0 Hz, 2H), 8.17-8.11 (m, 8H), 7.73 (d, J = 10.0 Hz, 4H), 7.61 (d, J = 5.0 Hz, 4H), 1.55-1.54 (m, 24H), 1.26-1.20 (m, 36H). ^{13}C NMR (125 MHz, acetone-d_6) δ 152.7, 146.0, 141.5, 137.2, 136.1, 133.5, 133.2, 129.8, 129.3, 129.2, 128.0, 127.0, 97.0, 84.3, 12.4, 12.3, 12.1, 7.0. ^{31}P NMR (acetone-d_6, 202 MHz): δ 14.54 (s, $J_{Pt\text{-}P}$= 2663.2 Hz).

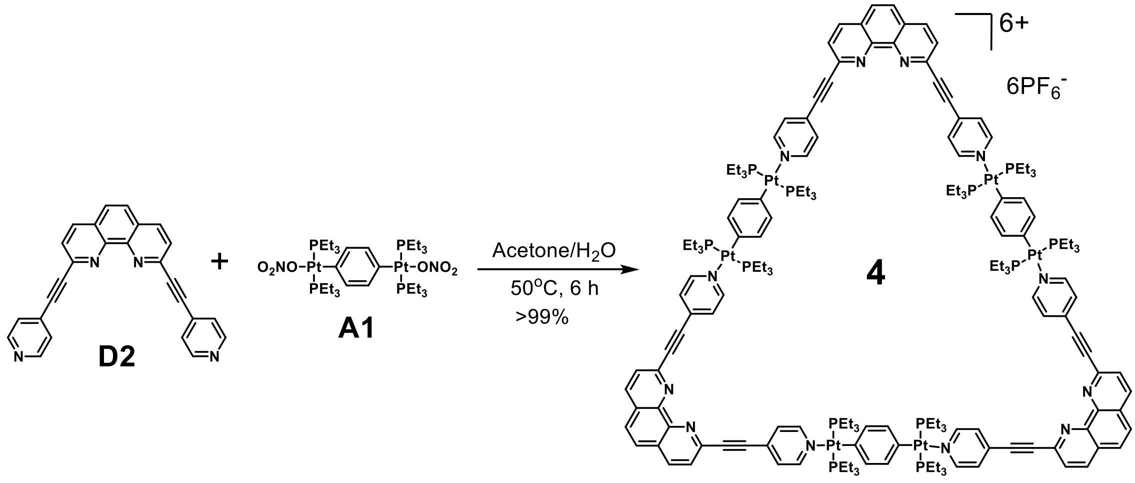

Supplementary Figure 5. The synthetic procedure for the metallacycle **4**.

*Synthesis of metallacycle **4***. Self-assembly of metallacycle **4** from donor **D2** and diplatinum acceptor **A1**. The dipyridyl donor ligand **D2** (5.67 mg, 14.9 μmol) and 180° organoplatinum acceptor **A1** (15.84 mg, 14.9 μmol) were weighed accurately into a glass vial. To the vial was added 2.0 mL acetone and 0.4 mL H_2O, and the reaction solution was stirred at room temperature for 6 hours. The PF_6^- salt of **4** was synthesized by dissolving the NO_3^- salt of **4** in acetone/H_2O and adding a saturated aqueous solution of KPF_6 to precipitate the product, which was collected by vacuum filtration. Yield: 23.77 mg, 99%. ^{1}H NMR (500 MHz, acetone-d_6) δ 9.13 (d, J = 5.0 Hz, 4H), 8.67 (d, J = 10.0 Hz, 2H), 8.16 (s, 2H), 8.14 (s, 2H), 8.09 (d, J = 5.0 Hz, 4H), 7.22 (s, 4H), 1.56-1.55 (m, 24H), 1.25-1.18 (m, 36H). ^{13}C

NMR (125 MHz, acetone-d_6) δ 152.7, 146.0, 141.6, 137.2, 136.6, 133.2, 129.6, 129.2, 128.0, 127.2, 96.8, 84.6, 12.5, 12.3, 12.2, 7.1. ^{31}P NMR (acetone-d_6, 202 MHz): δ 14.44 (s, J_{Pt-P}= 2719.7 Hz).

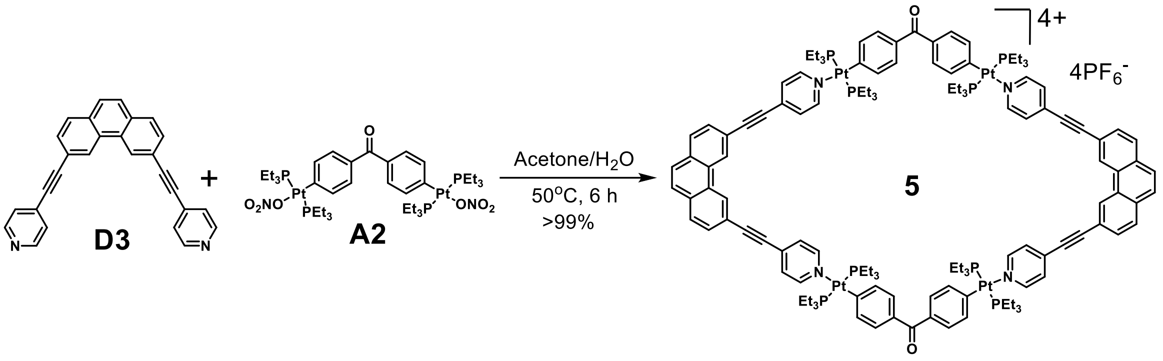

Supplementary Figure 6. The synthetic procedure for the metallacycle **5**.

Synthesis of metallacycle 5. Self-assembly of metallacycle **5** from donor **D3** and diplatinum acceptor **A2**. The dipyridyl donor ligand **D2** (4.49 mg, 11.8 μmol) and 120° organoplatinum acceptor **A2** (13.71 mg, 11.8 μmol) were weighed accurately into a glass vial. To the vial was added 2.0 mL acetone and 0.4 mL H_2O, and the reaction solution was stirred at room temperature for 6 hours. The PF_6^- salt of **5** was synthesized by dissolving the NO_3^- salt of **5** in acetone/H_2O and adding a saturated aqueous solution of KPF_6 to precipitate the product, which was collected by vacuum filtration. Yield: 20.01 mg, 99%. ^{1}H NMR (500 MHz, acetone-d_6) δ 9.29 (s, 2H), 9.10 (d, J = 5.0 Hz, 4H), 8.18 (d, J = 10.0 Hz, 2H), 8.06-8.02 (m, 6H), 7.93 (d, J = 5.0 Hz, 2H), 7.73 (d, J = 5.0 Hz, 4H), 7.61 (d, J = 10.0 Hz, 4H), 1.55-1.53 (m, 21H), 1.26-1.20 (m, 33H). ^{13}C NMR (125 MHz, acetone-d_6) δ 152.5, 136.1, 134.4, 133.1, 129.5, 129.3, 128.5, 119.9, 98.5, 86.3, 12.4, 12.3, 12.2, 7.0 (s). ^{31}P NMR (acetone-d_6, 202 MHz): δ 14.60 (s, J_{Pt-P}= 2656.3 Hz).

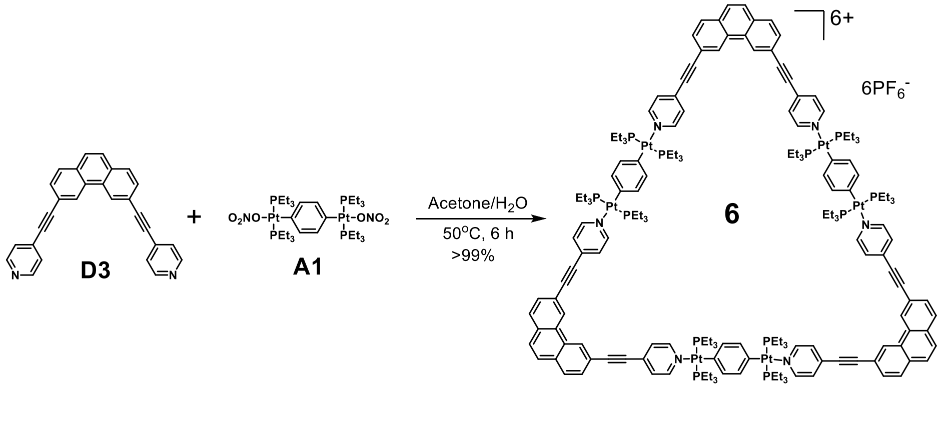

Supplementary Figure 7. The synthetic procedure for the metallacycle **6**.

*Synthesis of metallacycle **6***. Self-assembly of metallacycle **6** from donor **D3** and diplatinum acceptor **A1**. The dipyridyl donor ligand **D3** (3.80 mg, 10.0 μmol) and 120° organoplatinum acceptor **A1** (10.63 mg, 10.0 μmol) were weighed accurately into a glass vial. To the vial was added 2.0 mL acetone and 0.4 mL H_2O, and the reaction solution was stirred at room temperature for 6 hours. The PF_6^- salt of **6** was synthesized by dissolving the NO_3^- salt of **6** in acetone/H_2O and adding a saturated aqueous solution of KPF_6 to precipitate the product, which was collected by vacuum filtration. Yield: 15.93 mg, 99%. ^{1}H NMR (500 MHz, acetone-d_6) δ 9.27 (s, 2H), 9.07 (d, J = 5.0 Hz, 4H), 8.18 (d, J = 5.0 Hz, 2H), 8.05 (s, 2H), 8.02-7.96 (m, 4H), 7.94 (dd, 2H), 7.22 (s, 4H), 1.57-1.54 (m, 24H), 1.25-1.18 (m, 36H). ^{13}C NMR (125 MHz, acetone-d_6) δ 152.5, 136.6, 134.1, 133.1, 129.8, 129.6, 129.5, 129.1, 128.5, 127.9, 120.0, 98.2, 86.4, 12.5, 12.3, 12.2, 7.1. ^{31}P NMR (acetone-d_6, 202 MHz): δ 14.48 (s, $J_{Pt\text{-}P}$= 2733.5 Hz).

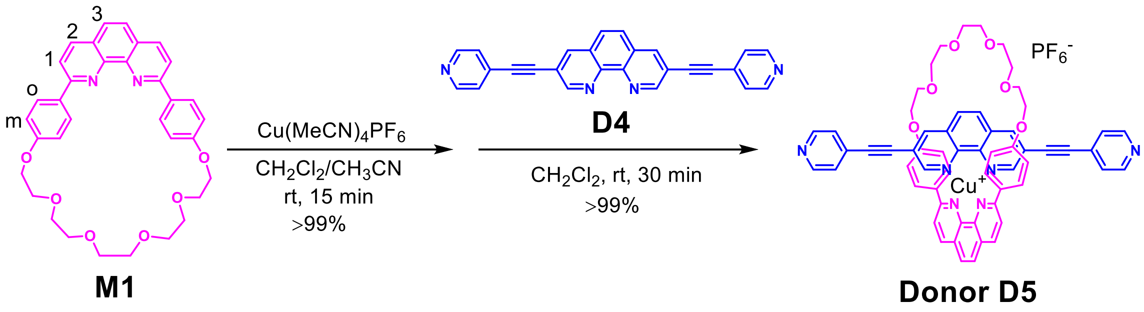

Supplementary Figure 8. The synthetic procedure for the pesudorotaxane-containing donor building block **D5**.

*Synthesis of donor **D5***. In a Schlenk flask, 1.0 equiv of macrocycle **M1** (52.22 mg, 0.092 mmol) was dissolved under argon in a 1:1 mixture of dichloromethane and acetonitrile. After addition of 1.0 equiv of $Cu(MeCN)_4PF_6$ (34.37 mg, 0.092 mmol), the reaction was stirred at room temperature under argon for 15 min. In a second Schlenk flask, 1.0 equiv of **D4** (35.18 mg, 0.092 mmol) was dissolved in dichloromethane and cannula-filtered into the first solution. The solution was stirred under argon at room temperature for an additional 30 min, followed by removal of the solvent in vacuo to dryness to afford quantitatively **D5** as a brown-red solid (yield = 106.48 mg, 99%). ^{1}H NMR (500 MHz, CD_2Cl_2) δ 8.77 (s, 2H), 8.68 (dd, J = 15.8, 6.7 Hz, 6H), 8.43 (s, 3H), 8.27 (s, 2H), 8.21-8.07 (m, 4H), 7.44 (dd, J = 18.0, 6.4 Hz, 8H), 6.00 (d, J = 7.9 Hz, 4H), 3.85 (s, 4H), 3.74 (dd, J = 11.7, 7.3 Hz, 5H), 3.62-3.55 (m, 5H), 3.48 (s, 8H). ESI-TOF-MS of **D5**: m/z calcd for [$C_{60}H_{48}N_6O_6CuPF_6$] ([M-PF_6^-]$^+$): 1011.63, Found: 1011.1.

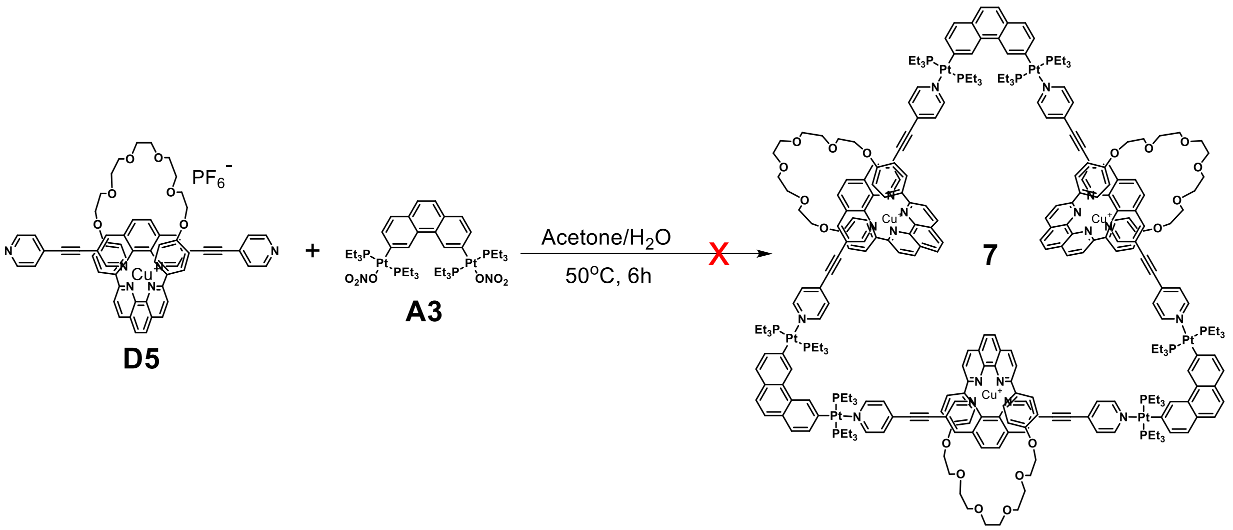

Supplementary Figure 9. Unsuccessful attempt for the synthesis of molecular necklace **7**.

Unsuccessful synthesis of molecular necklace ***7****.* The attempt to synthesize heterometallic triangular necklace **7** by the same protocol used to synthesize assemblies **1-6** was failed, indicated by the complicated ^{1}H NMR and ^{31}P NMR spectrum shown in Supplementary Figure 20.

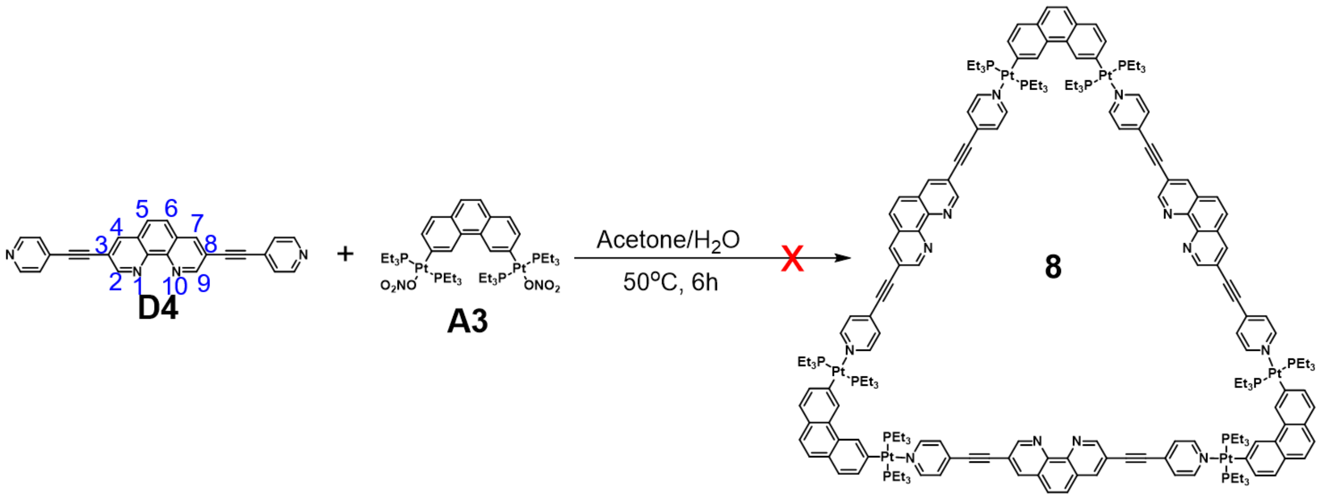

Supplementary Figure 10. Unsuccessful attempt for the synthesis of metallacycle **8**.

Unsuccessful synthesis of metallacycle ***8****.* Self-assembly of metallacycle **8** from donor **D4** and diplatinum acceptor **A3** was also unsuccessful, and the obtained product showed complicated ^{1}H NMR and ^{31}P NMR spectrum shown in Supplementary Figure 20.

2. Supplementary Note 1: Experimental details of DNA cleavage, bacterium-binding and antimicrobial tests

DNA cleavage experiments. Gel electrophoresis was run on a 1% (w/v) agarose gel at 60 V for 30 min and photographed by means of a UV transilluminator and WD-9415B gel documentation system (Beijing Liuyi Instrument Factory, P. R. China). The light irradiation was performed using a Xe lamp (CEL-HXF300 14V 50W) with an optical filter which provides visible light around at 450 nm. The light intensity was around 50 mW cm^{-2} and the distance is kept at 10 cm.

Binding of the molecules on the glass to the bacterial cells. To investigate the binding activity of the molecules to the bacterial cells, the pathogenic *Pseudomonas aeruginosa* PAO1 (obtained from ATCC) was overnight cultured in LB medium at 37 ˚C with shaking, washed with PBS (pH = 7.4), and suspended in PBS at an concentration of 1×10^8 cells/mL. The molecules were dissolved in acetone (100 μM), and then dropped on the glass and dried to form a membrane. The bacterial suspension was then dropped on the membrane and incubated at 37 ˚C for 10 min. The glass was then washed three times with PBS, followed by staining with 4',6-diamidino-2-phenylindole (DAPI, 5 mg/L, Sigma). After washing three times, the cells adhered on the molecular membrane was observed using confocal microscopy (FV300, Olympus, Japan). The fluorescence intensity of the DAPI was quantified using the Image J software to indicate the adhered bacterial cells.

DLS analysis of the molecule-bacterium co-incubation system. To evaluate the effect of the molecules on bacterial aggregation, the PAO1 cells (5×10^7 cells /mL) were mixed with the molecules (8 μM) in PBS, incubated at 37 ˚C for 10 min. The size distribution of the mixture was then examined using a laser light scattering spectrometer (BI-200SM) equipped with a digital correlator (TurboCorr).

LPS adsorption assays. Adsorption of the bacterial LPS (Yuanye, China) by the molecules were also tested glass. 200 μL of the molecule solutions (100 μM, in acetone) were fixed on the glass and dried. The LPS solution (500 mg/L) was then dropped on the molecular membrane. After 10 min of incubation at 37 °C, the solutions were transferred to determination of the unadhered LPS contents.

Anti-bacterial assays. To assess the anti-bacterial efficiency of the molecules, the bacterial cells were suspended in LB medium at an initial concentration of 5×10^7 cells /mL, and then added into 96-well plates. The molecules were further added to different final concentrations (0, 2, 4, 8, 16 μM). The

mixtures were cultured at 37 ˚C for 12 h, The mixtures were then 10-gradiently diluted using PBS, and plated on LB plate. After cultured for 24 h, colony forming units (CFU) were counted, and cell viability was calculated by the CFU of each treatment divided by that of the control. The mixtures were also stained by DAPI (5 mg/L) and propidium iodide (PI, 5 mg/L, Sigma) for 5 min, and then observed by fluorescent microscopy (Leica DM3000, Germany). Meanwhile, the cells were further fixed by 4% formaldehyde, followed by treatment of ethanol solution, and dried for scanning electron microscopy (SEM) observation (FEI Quanta 400 ESEM FEG).

ROS assays. To evaluate the intracellular ROS levels, the cells were treated by the molecules for 12 h, stained by 2',7'-dichlorodihydrofluorescein diacetate (DCFH-DA, 10 mg/L, Sigma) for 30 min. The fluorescence intensity of the cells (Excitation wavelength 488 nm, Emission wavelength 520 nm) was then measured using a fluorescence microplate reader (Enspire, Perkinelmer, USA).

Statistical analysis. Each experiment was performed in triplicate under each tested condition, and reported values represent the mean ± standard deviation. Significant difference ($P < 0.05$) between the treatments was determined using one-way ANOVA. All statistical analyses were performed using Statistical Packages for the Social Sciences (SPSS) Version 20.0.

3. Supplementary Note 2: Stability studies

The stabilities of necklace **1** and other assemblies under a variety of conditions including acid, base, tryptone, LB medium and chloride ion have been thoroughly surveyed by *in situ* ^{1}H NMR and ^{31}P NMR spectroscopy. All spectra were recorded in a mixed solvent of acetone-d_6 and D_2O (v/v 8:1) at 298 K. The degradation curve in Supplementary Figure 42c was obtained based on the integration values of ^{31}P NMR spectra in Supplementary Figure 42a and 42b.

4. Supplementary Tables

Supplementary Table 1. IC_{50} of the synthesized molecules against the standard and clinically isolated drug-resistant pathogens.

Molecules	IC_{50} (μM)			
	PAO1*	NKP2**	NKE2***	NKS1****
**1**	2.86 ± 0.23	3.98 ± 0.54	1.84 ± 0.27	5.16 ± 0.27
**2**	6.82 ± 1.17	8.19 ± 2.35	5.62 ± 1.40	14.32 ± 1.83
**3**	9.65 ± 2.56	12.63 ± 2.14	10.58 ± 1.86	24.35 ± 3.12
**4**	17.89 ± 2.93	22.39 ± 3.08	15.90 ± 2.34	29.80 ± 2.31
**5**	10.23 ± 1.31	15.45± 2.36	12.87 ± 2.58	17.59 ± 3.08
**6**	20.84 ± 2.50	35.68 ± 3.30	16.89 ± 2.30	32.34 ± 2.87
**D1**	7.01 ± 0.66	10.45 ± 0.96	10.95 ±1.67	19.85 ± 1.82
$Cu(MeCN)_4PF_6$	7.98 ± 1.24	11.50 ± 2.28	11.85 ± 1.83	21.47 ± 2.59
**A1**	24.14 ± 1.78	52.32 ± 6.48	23.51 ± 2.60	36.40 ± 3.18
**A2**	31.12 ± 2.92	63.28 ± 4.50	29.86 ± 3.24	39.56 ± 3.42

Note: The strain PAO1 (*) is the standard strain of *P. aeruginosa*; the strain NKP2 (**) is a clinically isolated *P. aeruginosa* strain resistant to ciprofloxacin and penicillin; the strain NKE2 (***) is a clinically isolated *Escherichia coli* strain resistant to penicillin and tetracycline; the strain NKS1 (****) is a clinically isolated multidrug-resistant *Staphylococcus aureus* (MRSA) strain resistant to ciprofloxacin and penicillin.

5. Supplementary Figures

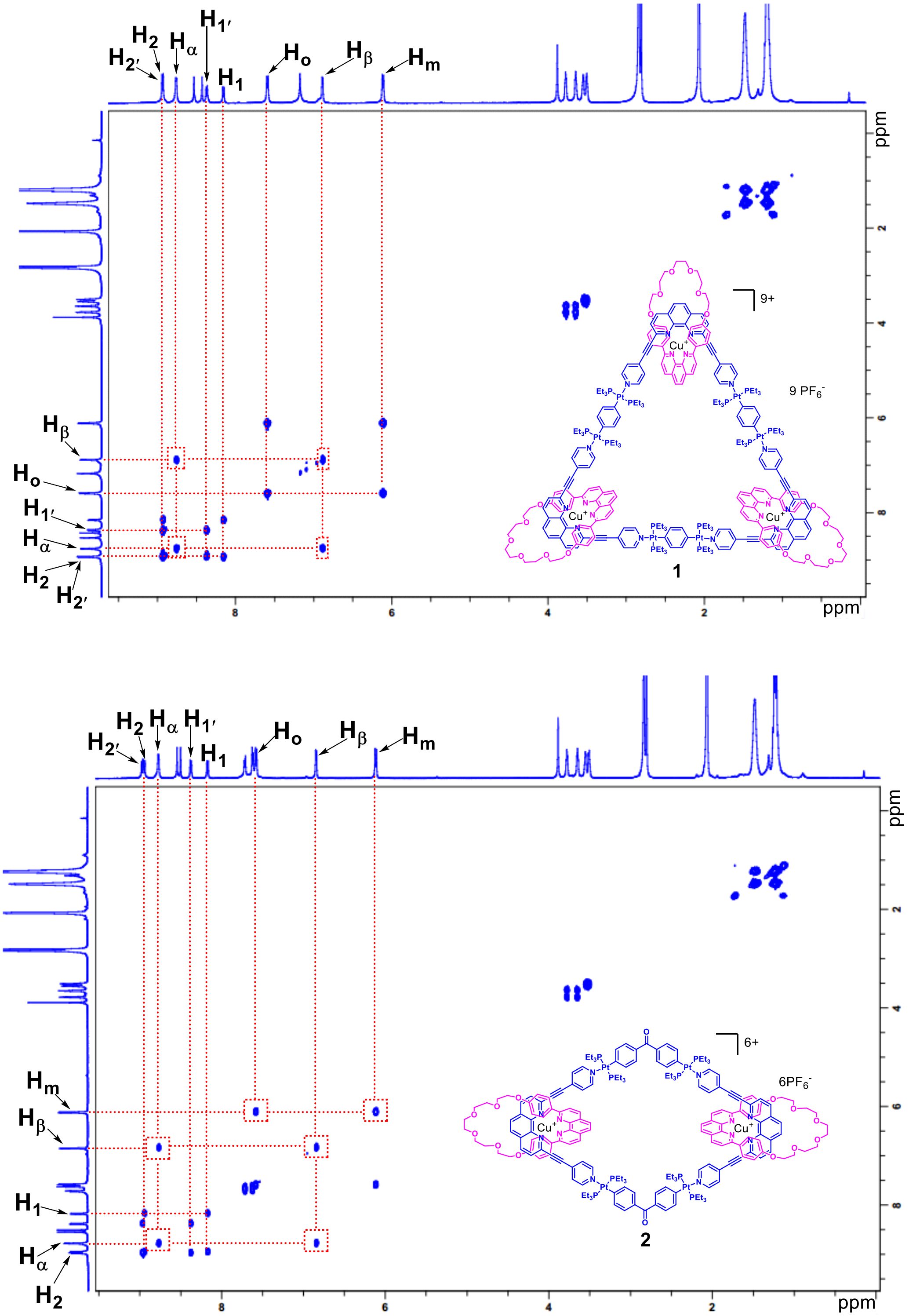

Supplementary Figure 11. ^{1}H-^{1}H COSY NMR spectra of molecular necklace **1** and [3]catenane **2** in acetone-d_6 (500 MHz, 296 K).

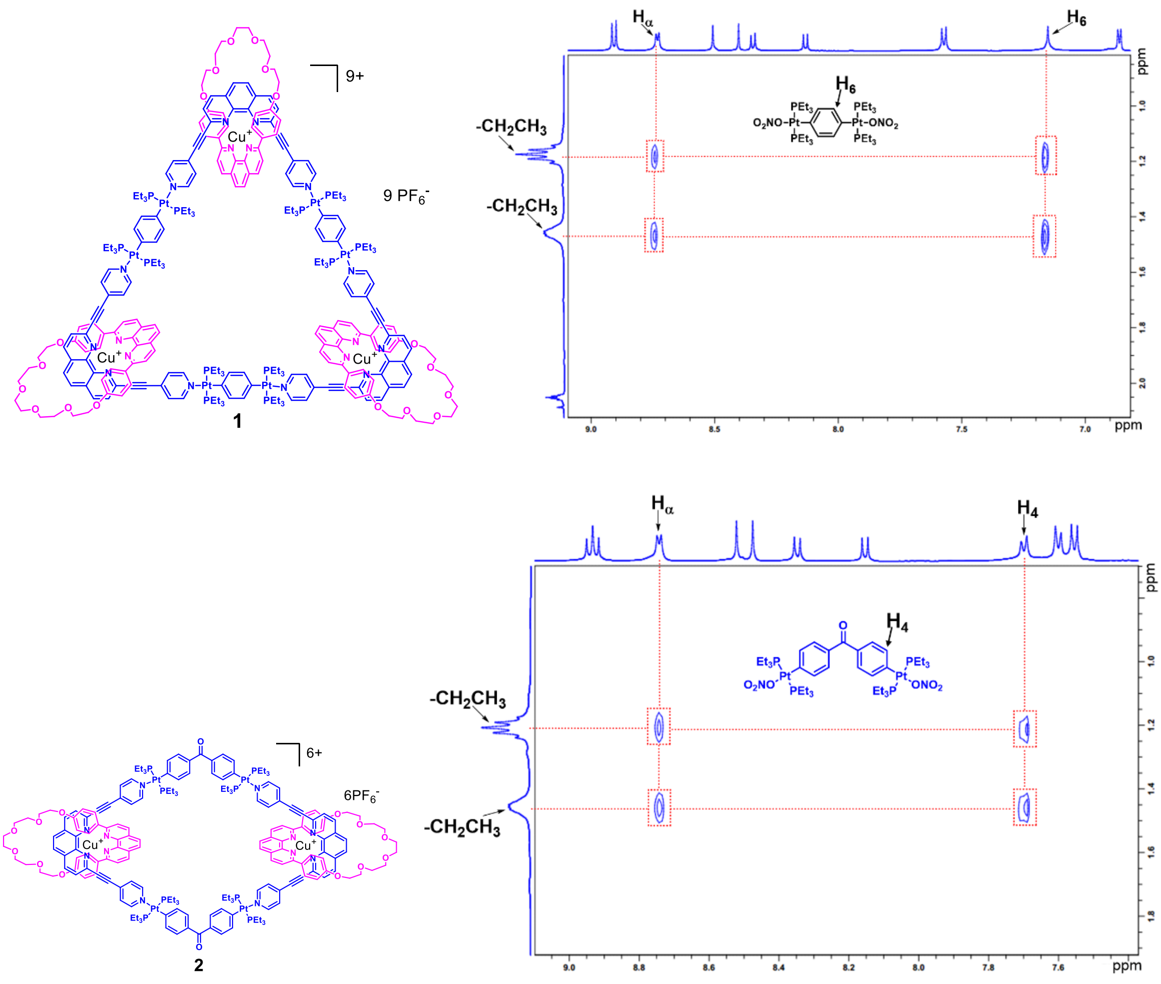

Supplementary Figure 12. 2D NOESY NMR spectra of molecular necklaces **1** and [3]catenane **2** in acetone-d_6 (500 MHz, 296 K).

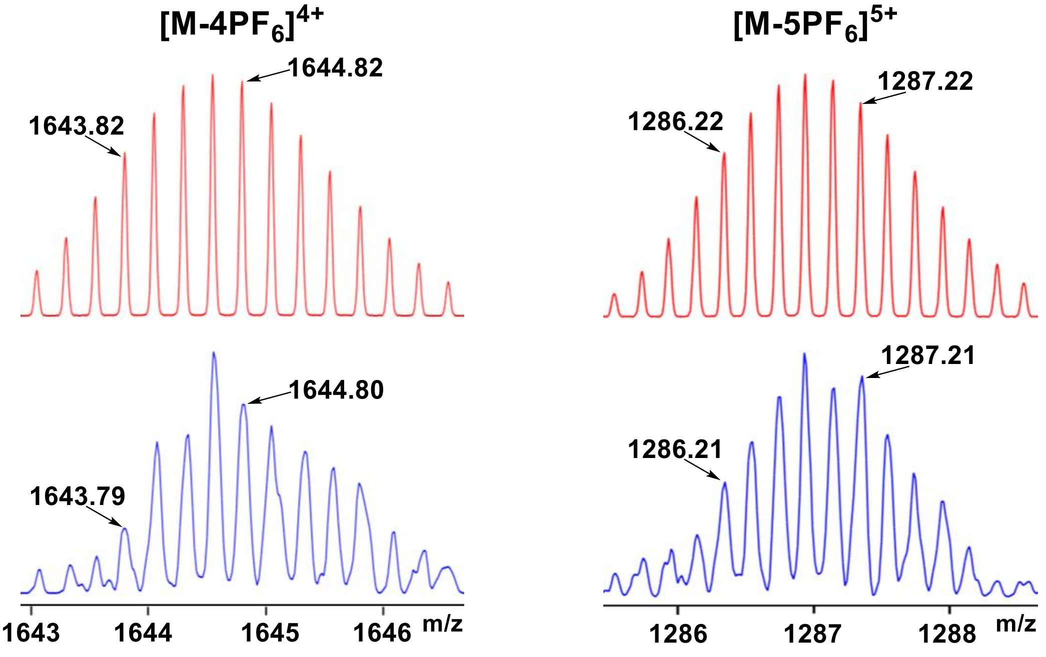

Supplementary Figure 13. Theoretical (top) and experimental (bottom) ESI-TOF-MS spectra of molecular necklace **1**.

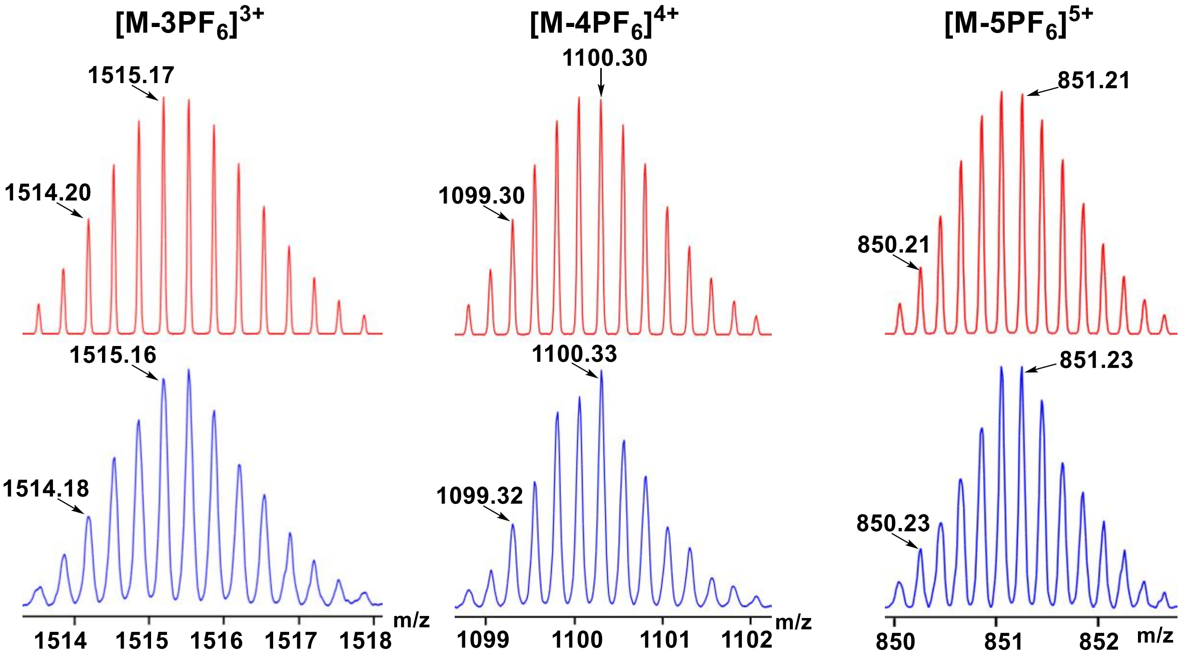

Supplementary Figure 14. Theoretical (top) and experimental (bottom) ESI-TOF-MS spectra of [3]catenane **2**.

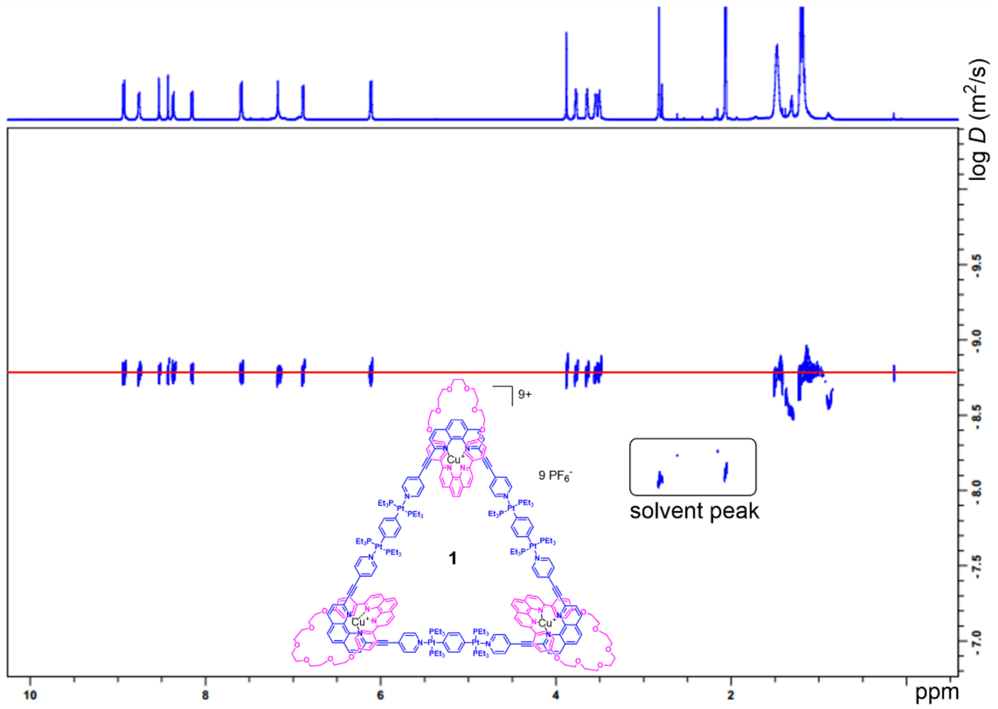

Supplementary Figure 15. 2-D DOSY spectrum (acetone-d_6, 298 K, 500 MHz) of molecular necklace **1**.

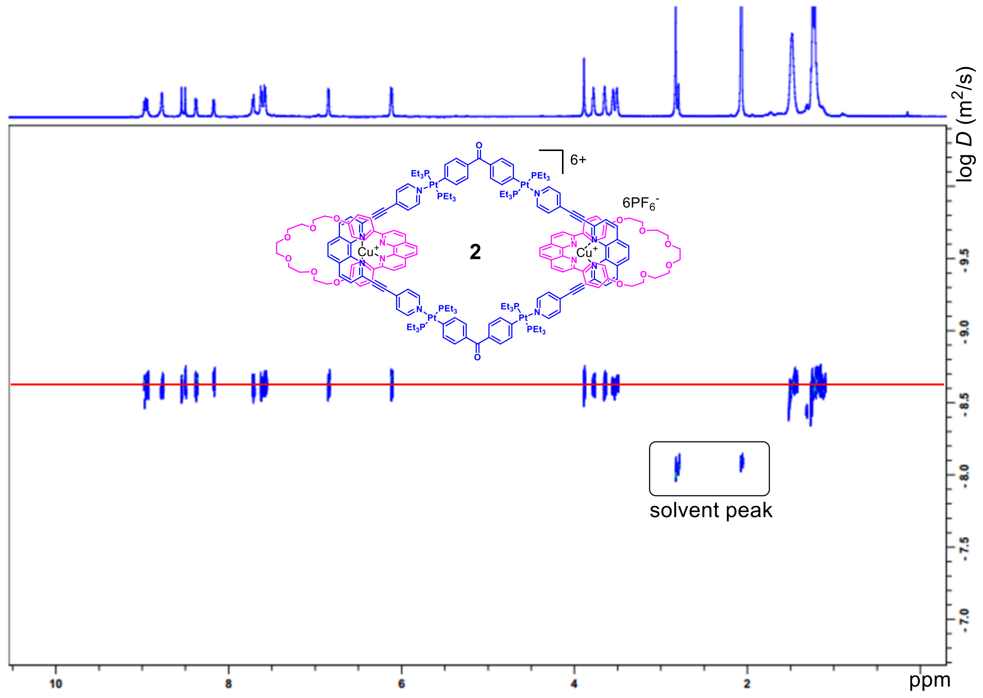

Supplementary Figure 16. 2-D DOSY spectrum (acetone-d_6, 298 K, 500 MHz) of [3]catenane **2**.

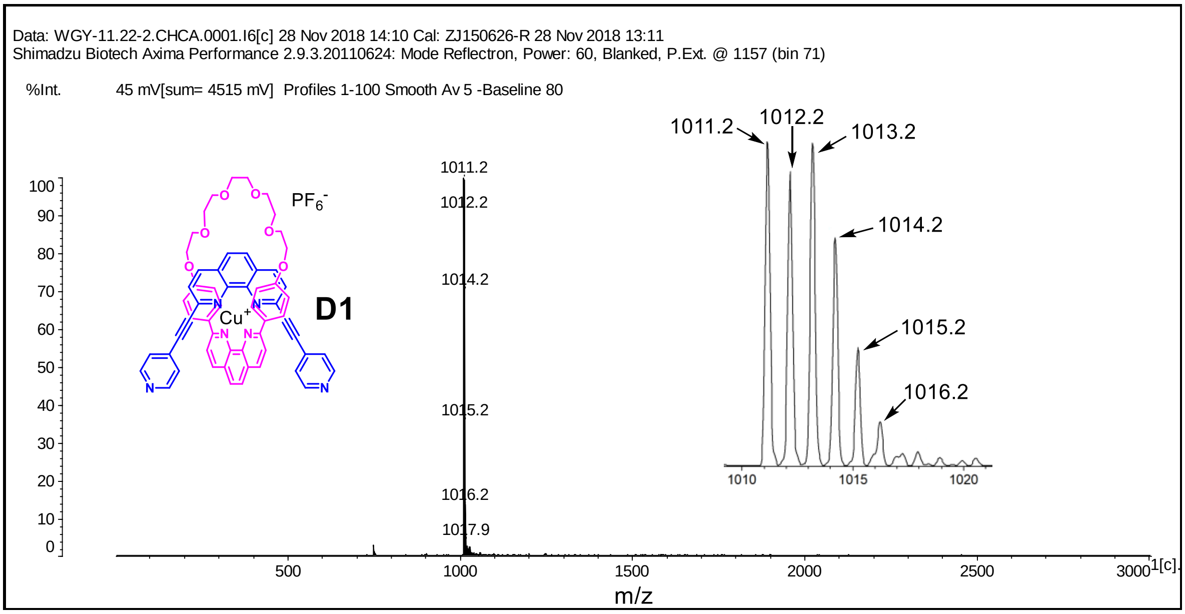

Supplementary Figure 17. ESI-TOF-MS of **D1**: Exact mass calcd. For $[C_{60}H_{48}N_6O_6Cu]^+$: 1012.63, Found: 1012.2.

5

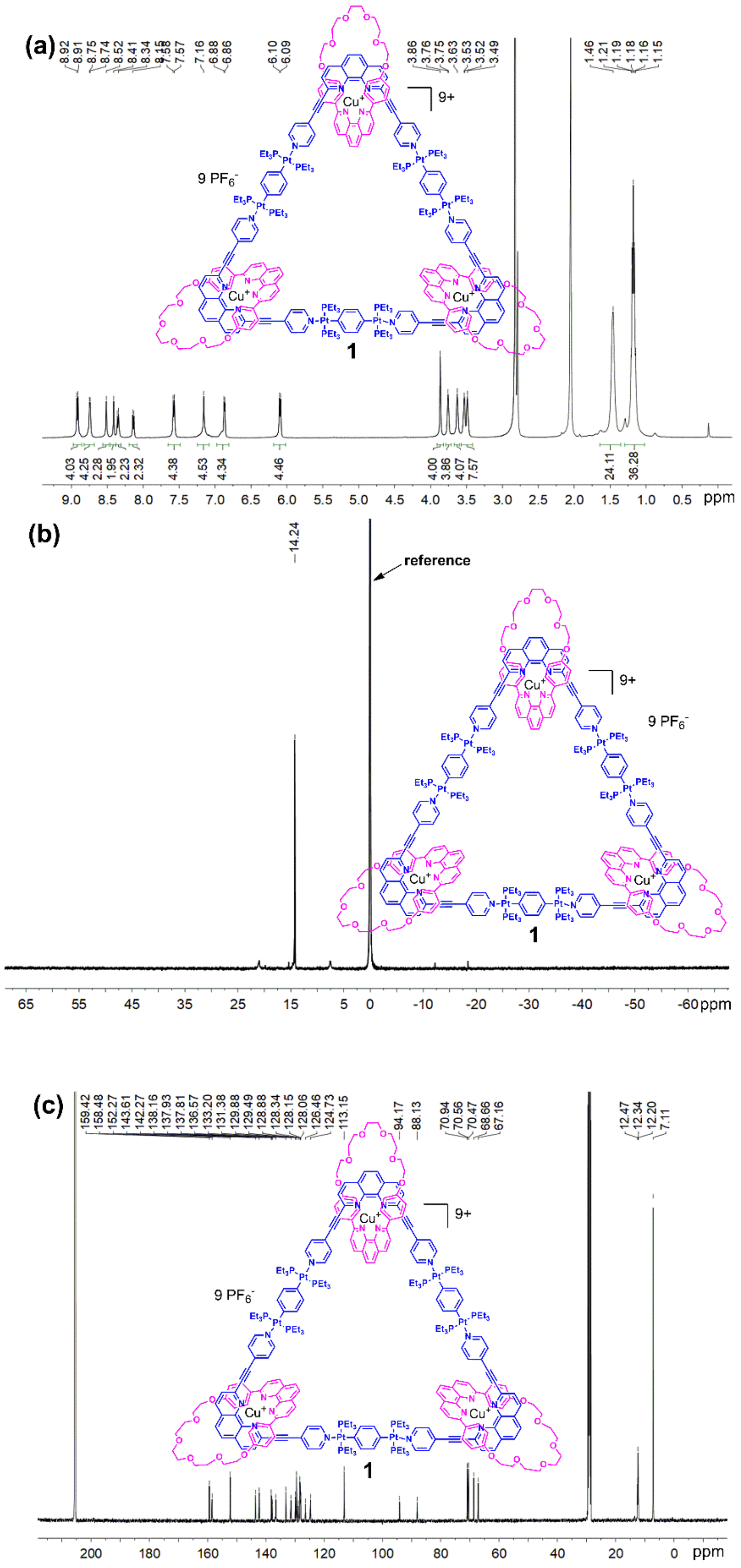

Supplementary Figure 18. (a) ^{1}H NMR spectrum (500 MHz, acetone-d_6, 298K), (b) ^{31}P NMR spectrum (202 MHz, acetone-d_6, 298K) and (c) ^{13}C NMR spectrum (125 MHz, acetone-d_6, 298K) of **1**.

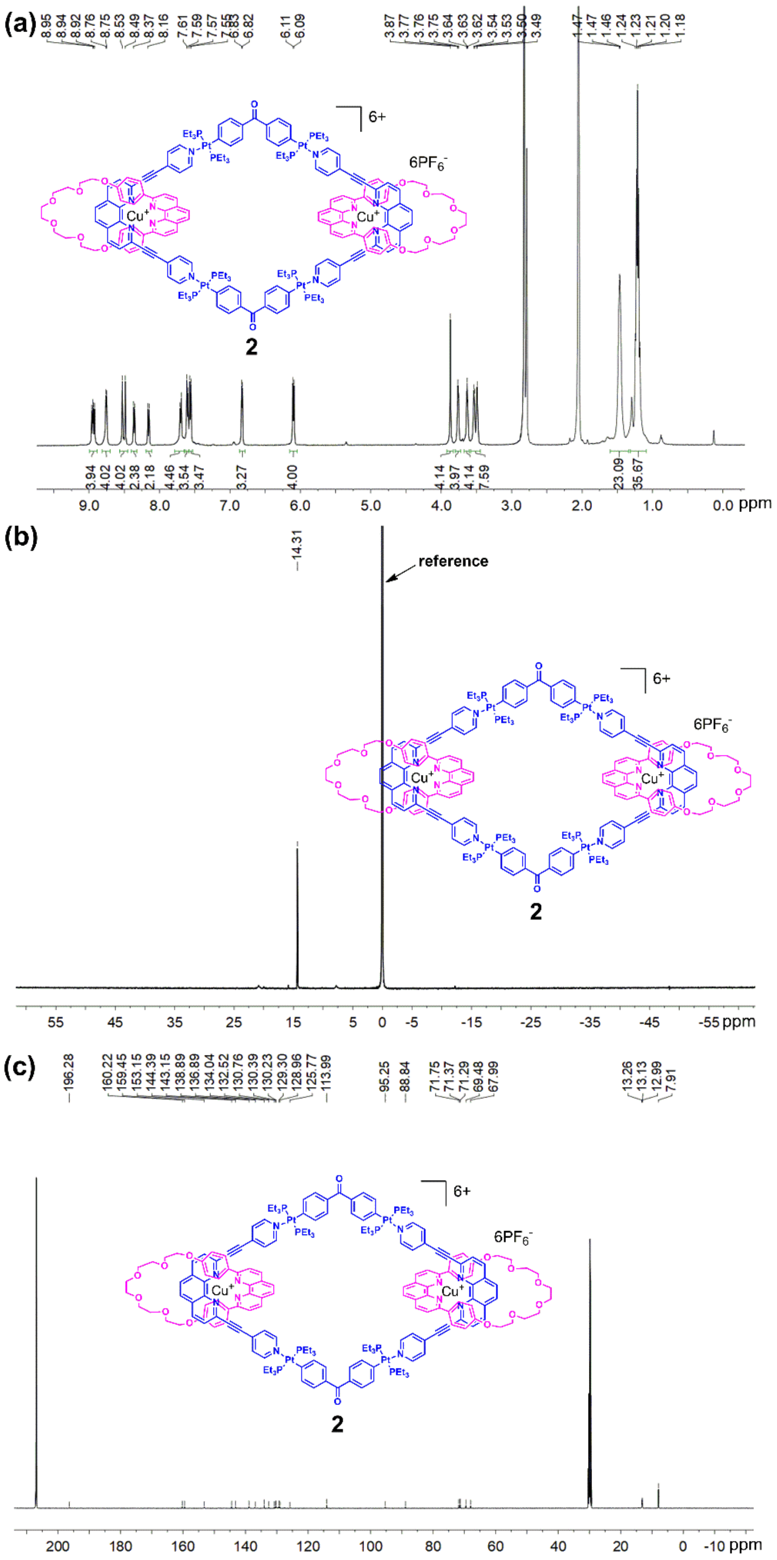

Supplementary Figure 19. (a) ^{1}H NMR spectrum (500 MHz, acetone-*d*$_6$, 298K), (b) ^{31}P NMR spectrum (202 MHz, acetone-*d*$_6$, 298K) and (c) ^{13}C NMR spectrum (125 MHz, acetone-*d*$_6$, 298K) of **2**.

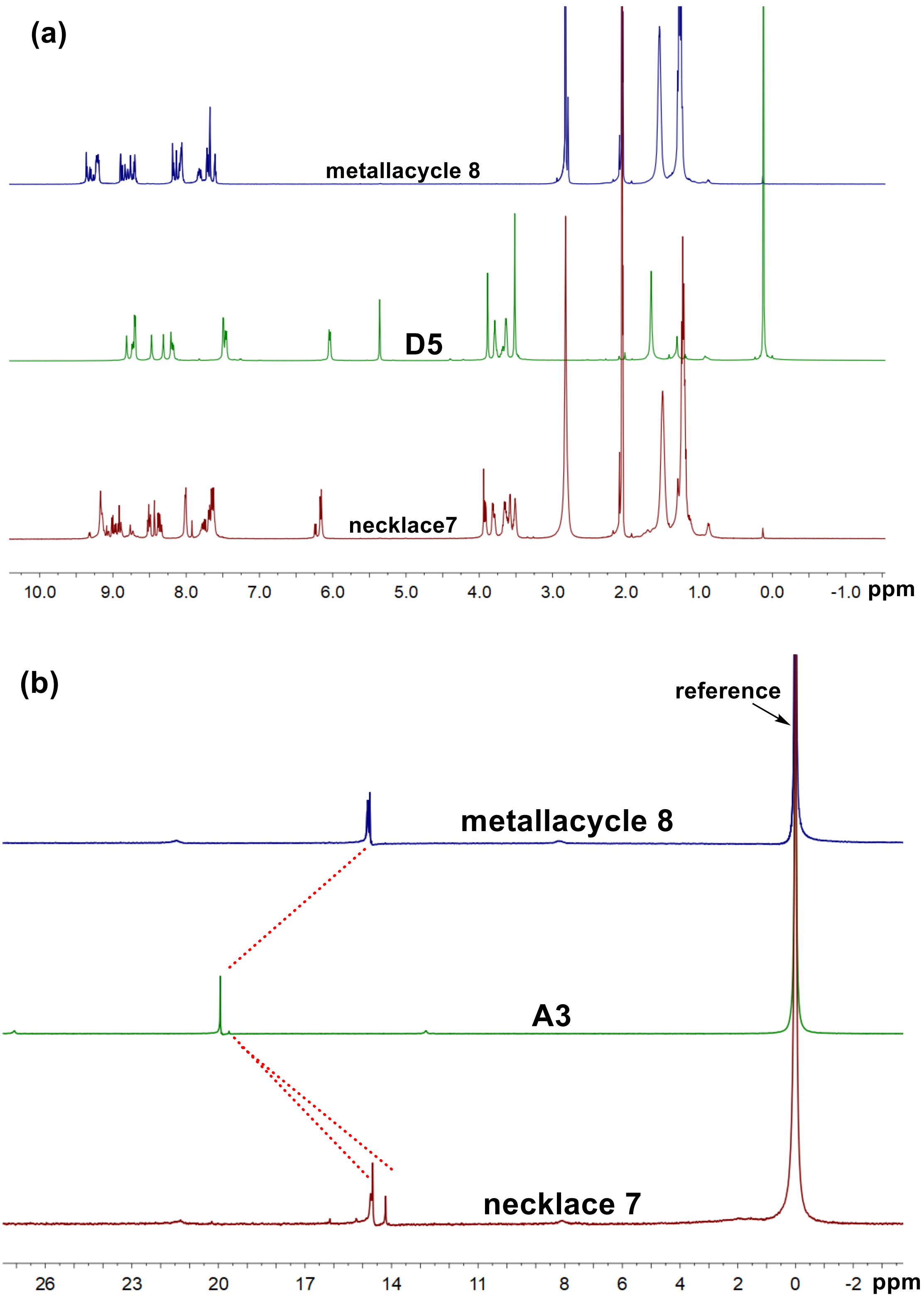

Supplementary Figure 20. (a) ^{1}H NMR spectrum (500 MHz, acetone-d_6, 298K), (b) ^{31}P NMR spectrum (202 MHz, acetone-d_6, 298K) of metallacycle **7** and **8**.

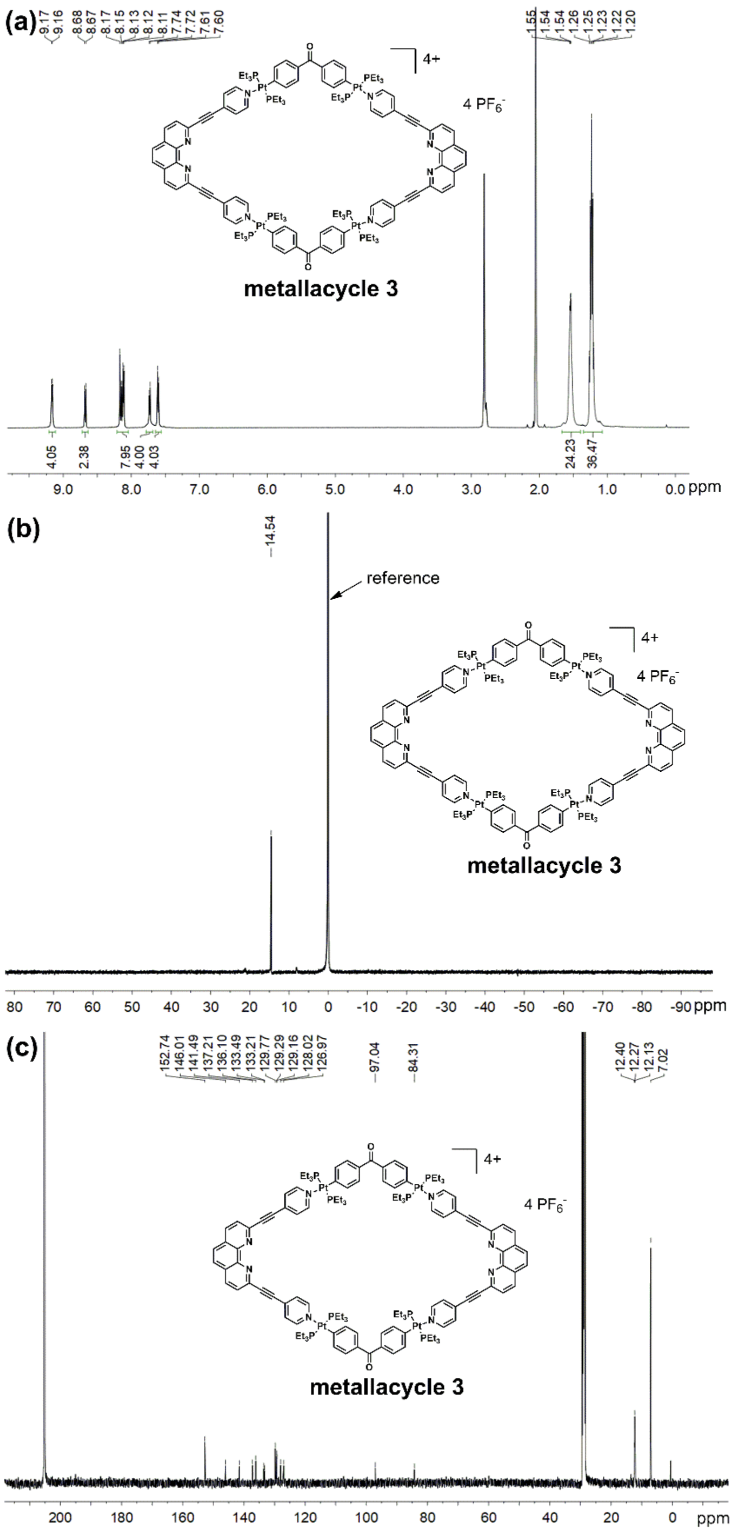

Supplementary Figure 21. (a) ^{1}H NMR spectrum (500 MHz, acetone-*d*$_6$, 298K), (b) ^{31}P NMR spectrum (202 MHz, acetone-*d*$_6$, 298K) and (c) ^{13}C NMR spectrum (125 MHz, acetone-*d*$_6$, 298K) of metallacycle **3**.

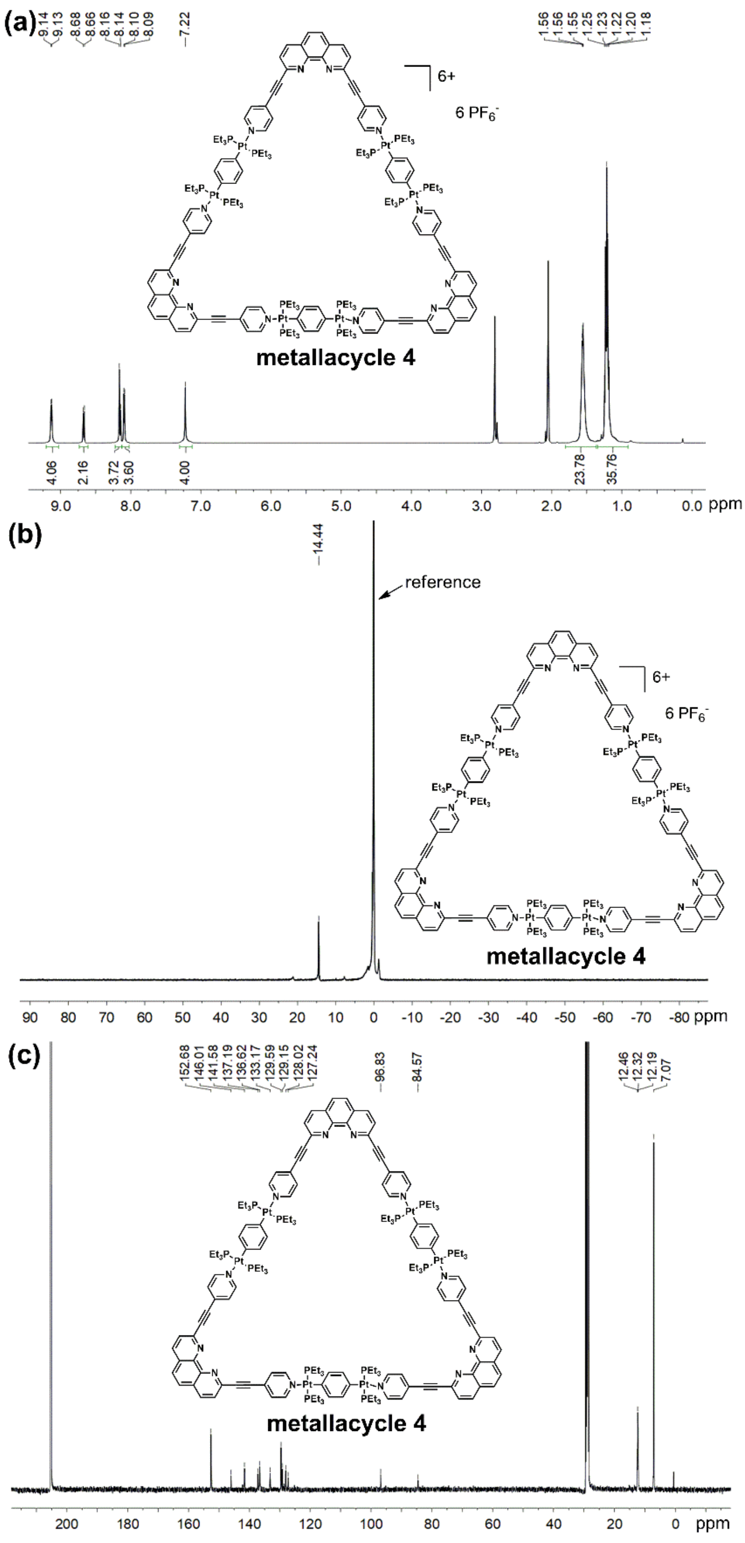

Supplementary Figure 22. (a) ^{1}H NMR spectrum (500 MHz, acetone-d_6, 298K), (b) ^{31}P NMR spectrum (202 MHz, acetone-d_6, 298K) and (c) ^{13}C NMR spectrum (125 MHz, acetone-d_6, 298K) of metallacycle **4**.

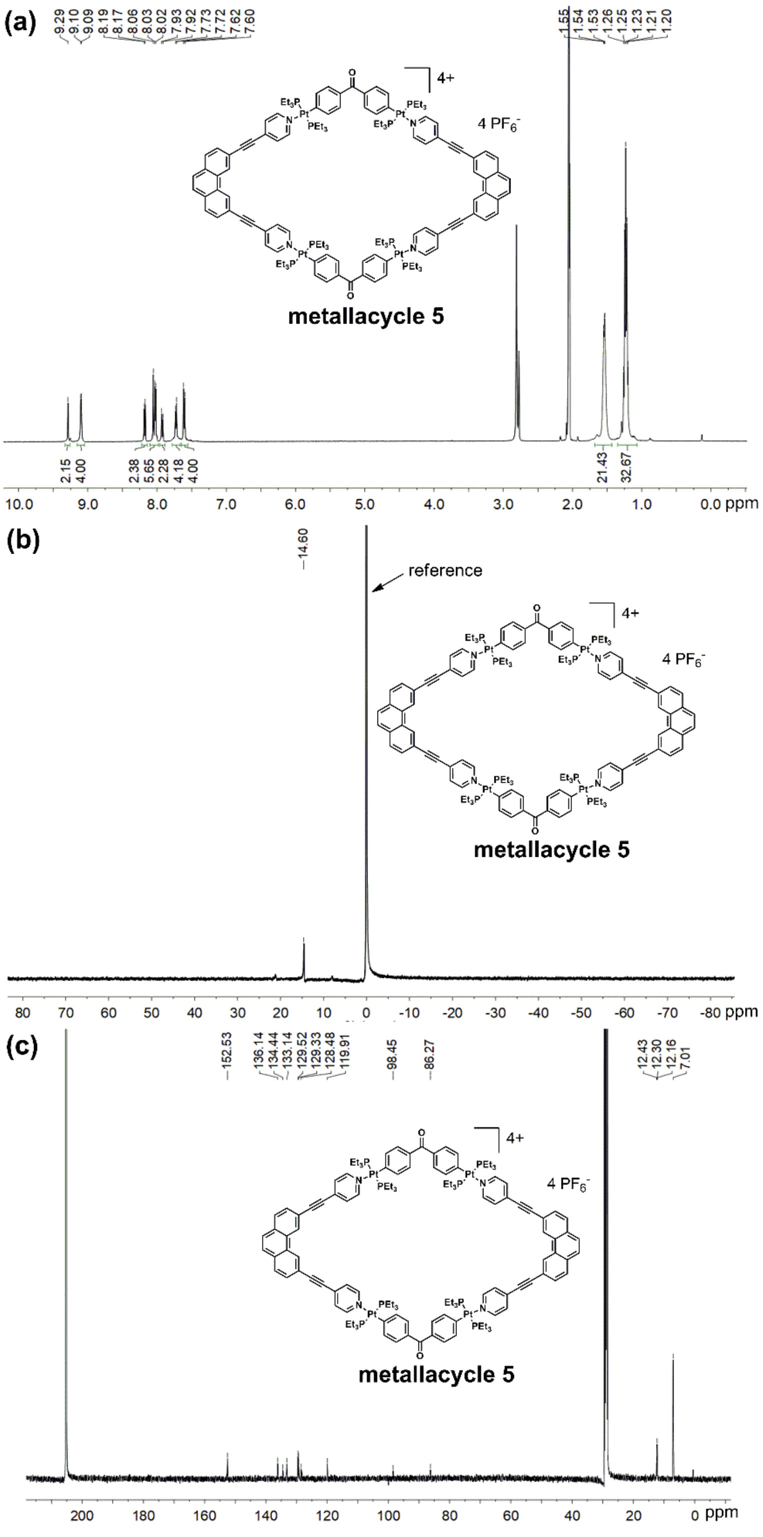

Supplementary Figure 23. (a) ^{1}H NMR spectrum (500 MHz, acetone-d_6, 298K), (b) ^{31}P NMR spectrum (202 MHz, acetone-d_6, 298K) and (c) ^{13}C NMR spectrum (125 MHz, acetone-d_6, 298K) of metallacycle **5**.

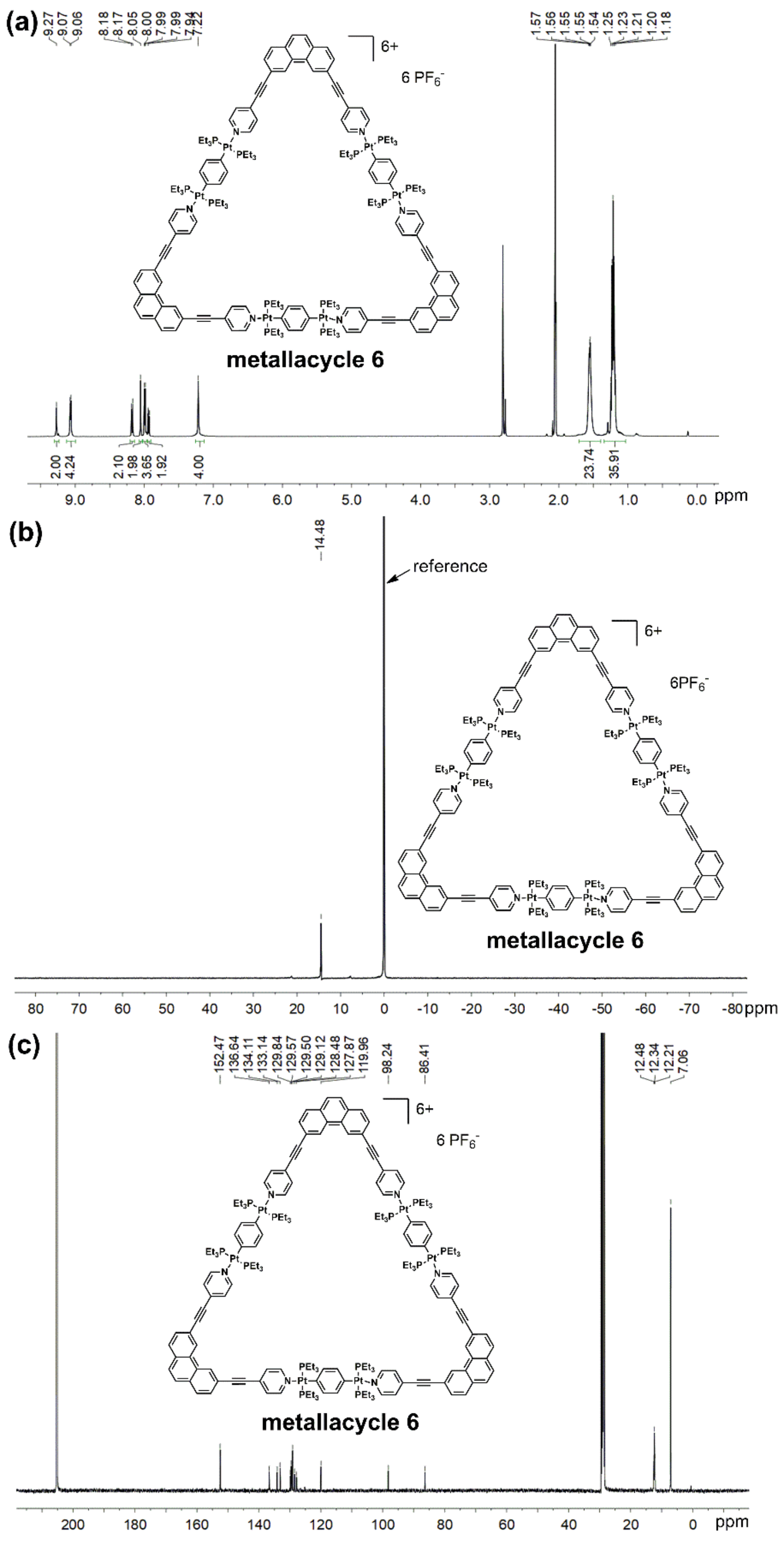

Supplementary Figure 24. (a) ^{1}H NMR spectrum (500 MHz, acetone-d_6, 298K), (b) ^{31}P NMR spectrum (202 MHz, acetone-d_6, 298K) and (c) ^{13}C NMR spectrum (125 MHz, acetone-d_6, 298K) of metallacycle **6**.

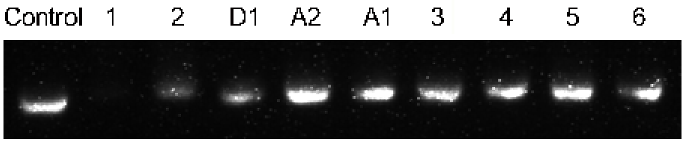

Supplementary Figure 25. Agarose gel electrophoresis patterns for the cleavage of pBR322 DNA by the molecular necklace **1**, [3]catenane **2**, the building blocks (**D1, A2, A1**), and the metallacycles (**3, 4, 5, 6**). Conditions: 25 ng/μL DNA; 6 μM building block and molecular necklaces; incubations times: 10 min.

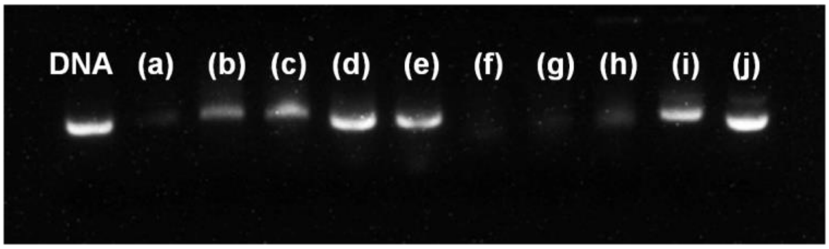

Supplementary Figure 26. Dose-dependent DNA cleavage by molecular necklaces **1** and [3]catenane **2**. Agarose gel electrophoresis patterns for the cleavage of pBR322 DNA by **2 (a-e)** and **1 (f-j)** in different concentrations. Conditions: 25 ng/μL DNA; incubations times: 10 min. Lane DNA, Control DNA sample with no addition of the necklaces. Lane (a)-(e), DNA and **2** with different concentrations, 10 μM, 8 μM, 6 μM, 4 μM, 2 μM, respectively. Lane (f)-(j), DNA and **1** with different concentrations, 10 μM, 8 μM, 6 μM, 4 μM, 2 μM, respectively. incubations times: 10 min.

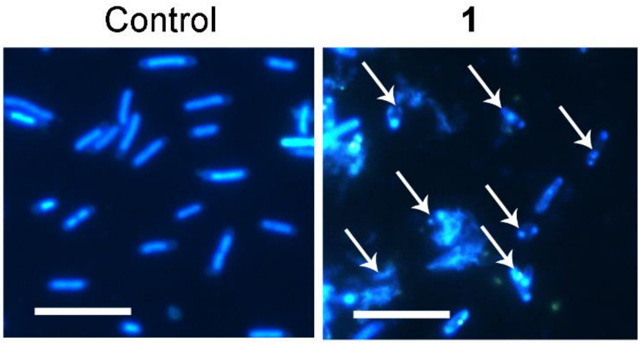

Supplementary Figure 27. DNA fragmentation in the *Pseudomonas aeruginosa* cells induced by the necklace **1** (Scale bar = 10 μm). The white arrows indicate intracellular DNA fragmentation induced by necklace **1**.

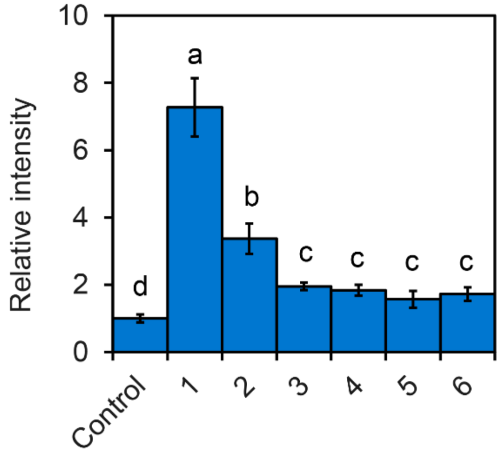

Supplementary Figure 28. Statistical analysis of the DAPI fluorescence intensity of the *P. aeruginosa* cells adhered on the surfaces of the molecular necklaces or the metallacycles. Data are represented as mean ± SD (n = 3). Different letters above the columns indicate significant difference between the groups ($P < 0.05$).

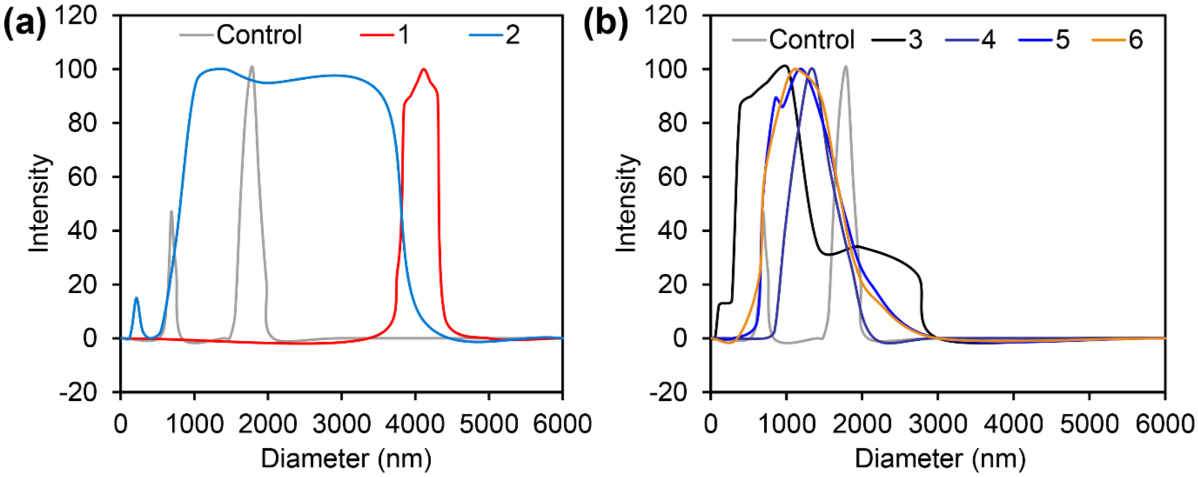

Supplementary Figure 29. Size distribution of the *P. aeruginosa*-necklace/metallacycle mixtures revealed by DLS. (a) Size distribution of the mixtures of the bacteria and the molecular necklaces **1** and [3]catenane **2**, noting that **1** results in more size increase than **2**. (b) Size distribution of the mixtures of the bacteria and the metallacycles **3, 4, 5** or **6**.

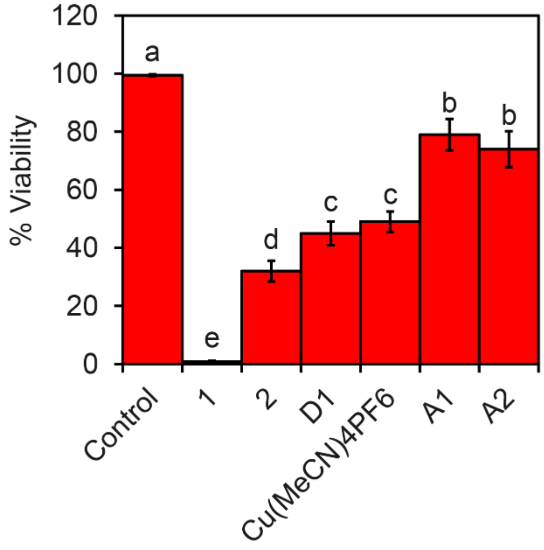

Supplementary Figure 30. Cell viability of the groups treated by the necklace **1**, **2**, the donor **D1,** Cu(MeCN)$_4$PF$_6$ (the precursor of **D1**) or the acceptors (**A1**, **A2**) (8 μM). Data are represented as mean ± SD (n = 3). Different letters above the columns indicate significant difference between the groups ($P < 0.05$).

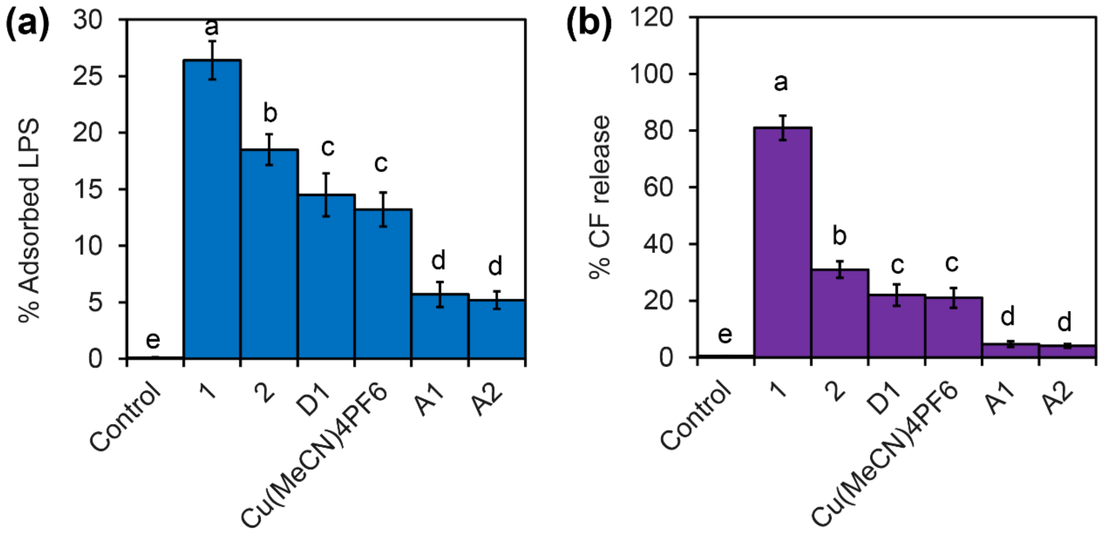

Supplementary Figure 31. LPS adsorption (a) and liposome damage (b) by the necklace **1**, **2**, the donor **D1,** Cu(MeCN)$_4$PF$_6$ (the precursor of **D1**) or the acceptor (**A1**, **A2**). Data are represented as mean ± SD (n = 3). Different letters above the columns indicate significant difference between the groups ($P < 0.05$).

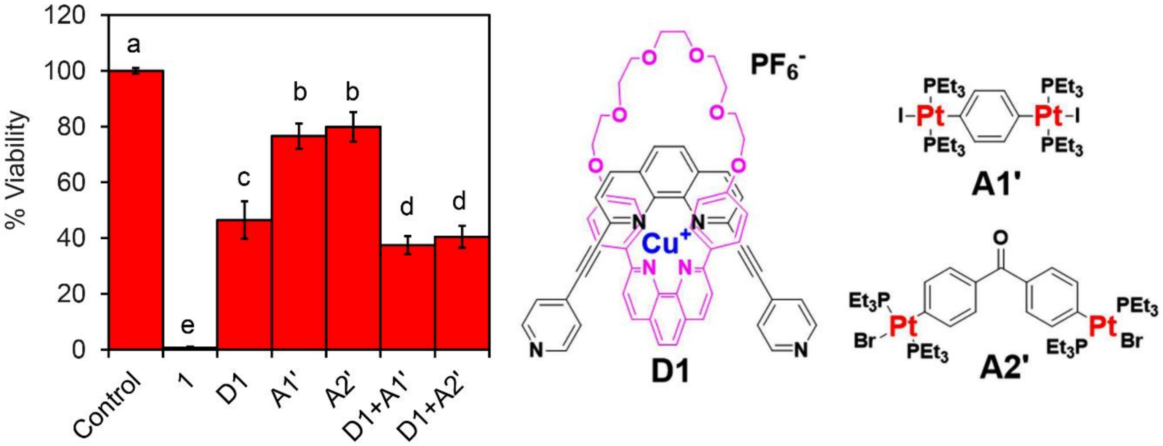

Supplementary Figure 32. Cell viability of the groups treated by the necklace **1** (8 μM), the donor **D1** (8 μM), the analogue of **A1** (**A1’**, 8 μM), the **A2** analogue of (**A2’**, 8 μM**)**, the mixture of **D1** and **A1’** (**D1+A1’**, 8 μM + 8 μM), and the mixture of **D1** and **A2’** (**D1+A2’**, 8 μM + 8 μM). Data are represented as mean ± SD (n = 3). Different letters above the columns indicate significant difference between the groups ($P < 0.05$).

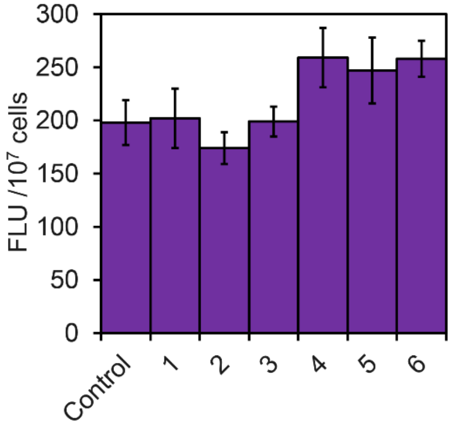

Supplementary Figure 33. ROS levels of the bacterial cells treated by the molecular necklace **1**, [3]catenane **2** or the metallacycles **3~6.** The intracellular ROS levels were evaluated by DCFH-DA staining, followed by fluorescence intensity measurement. Data are represented as mean ± SD (n = 3). Noting that there is no significant difference in intracellular ROS levels between the groups ($P < 0.05$).

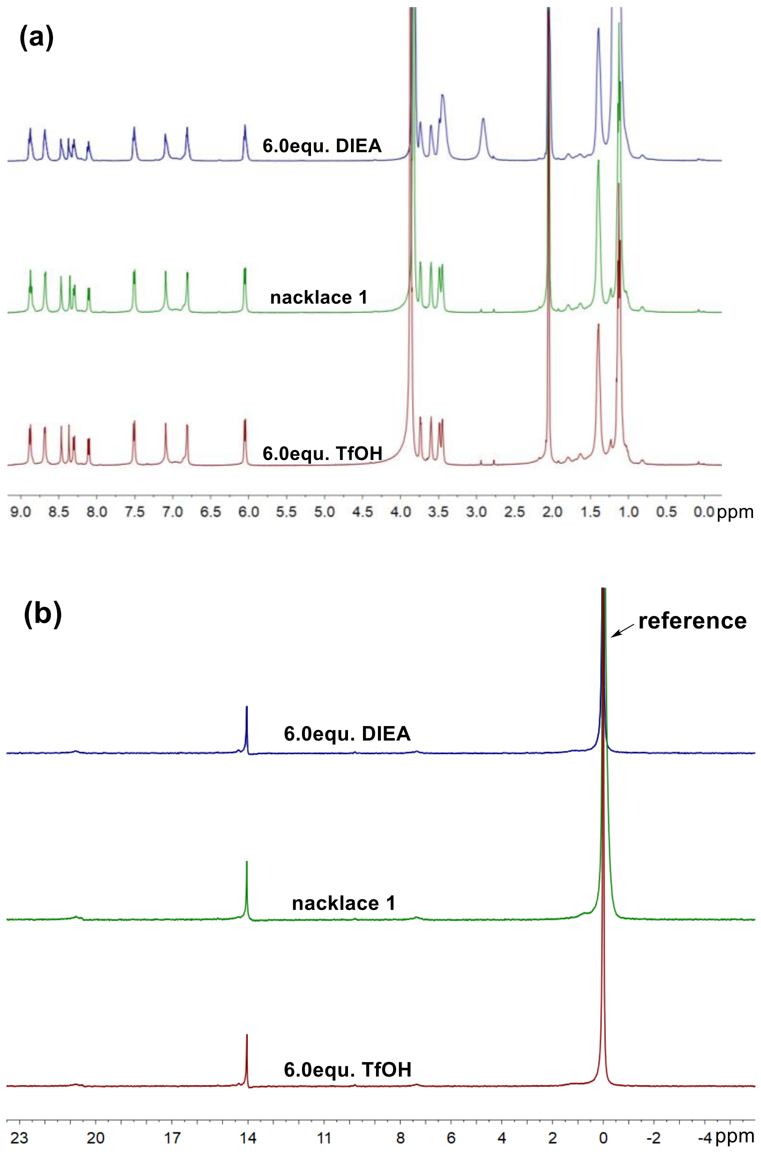

Supplementary Figure 34. (a) The stacked ^{1}H NMR spectra of molecular necklace **1** before and after adding N,N-Diisopropylethylamine (DIEA) or triflic acid (TfOH). (b) The stacked ^{31}P NMR spectra of molecular necklace **1** before and after adding DIEA or TfOH.

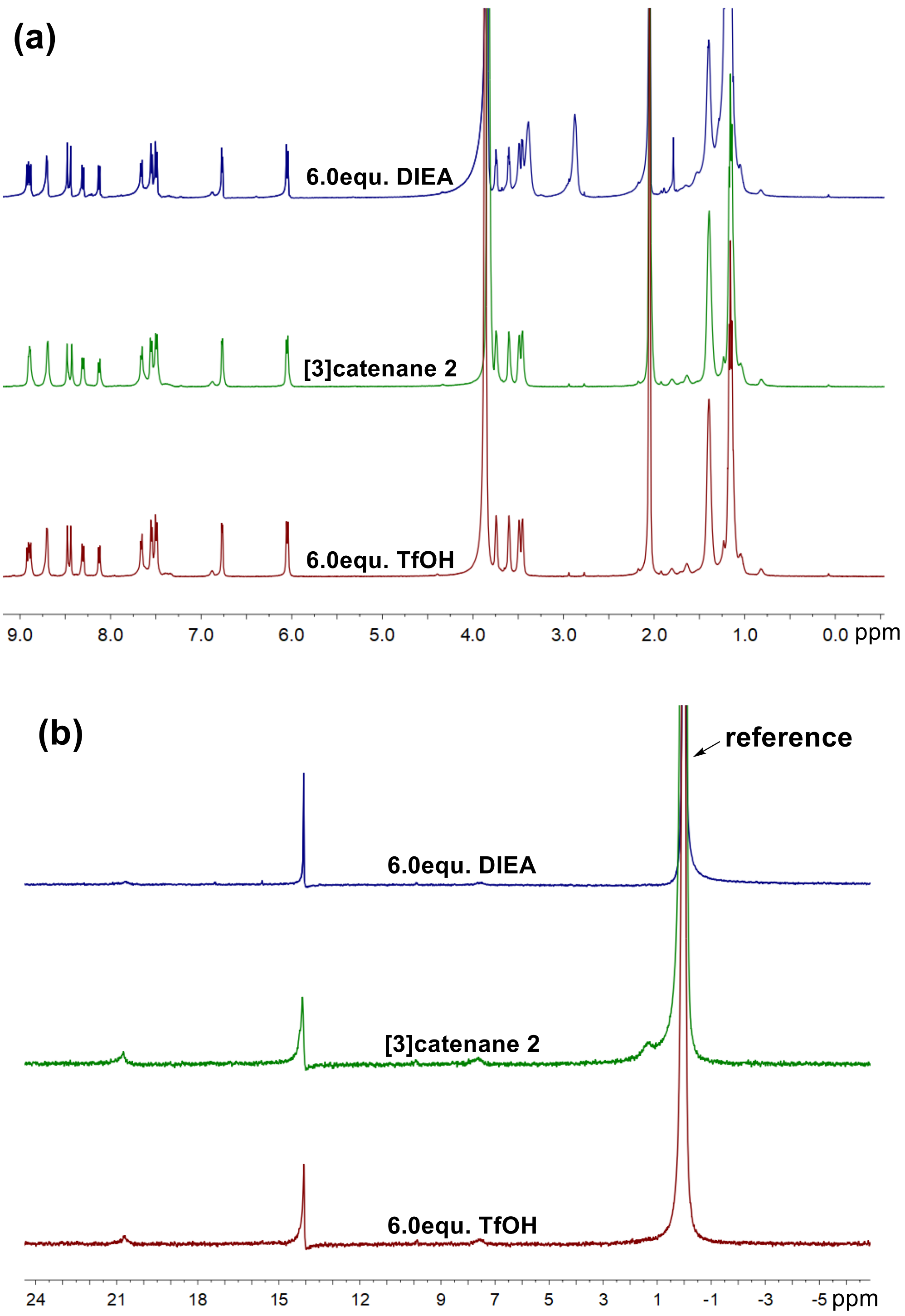

Supplementary Figure 35. (a) The stacked ^{1}H NMR spectra of [3]catenane **2** before and after adding DIEA or TfOH. (b) The stacked ^{31}P NMR spectra of [3]catenane **2** before and after adding DIEA or TfOH.

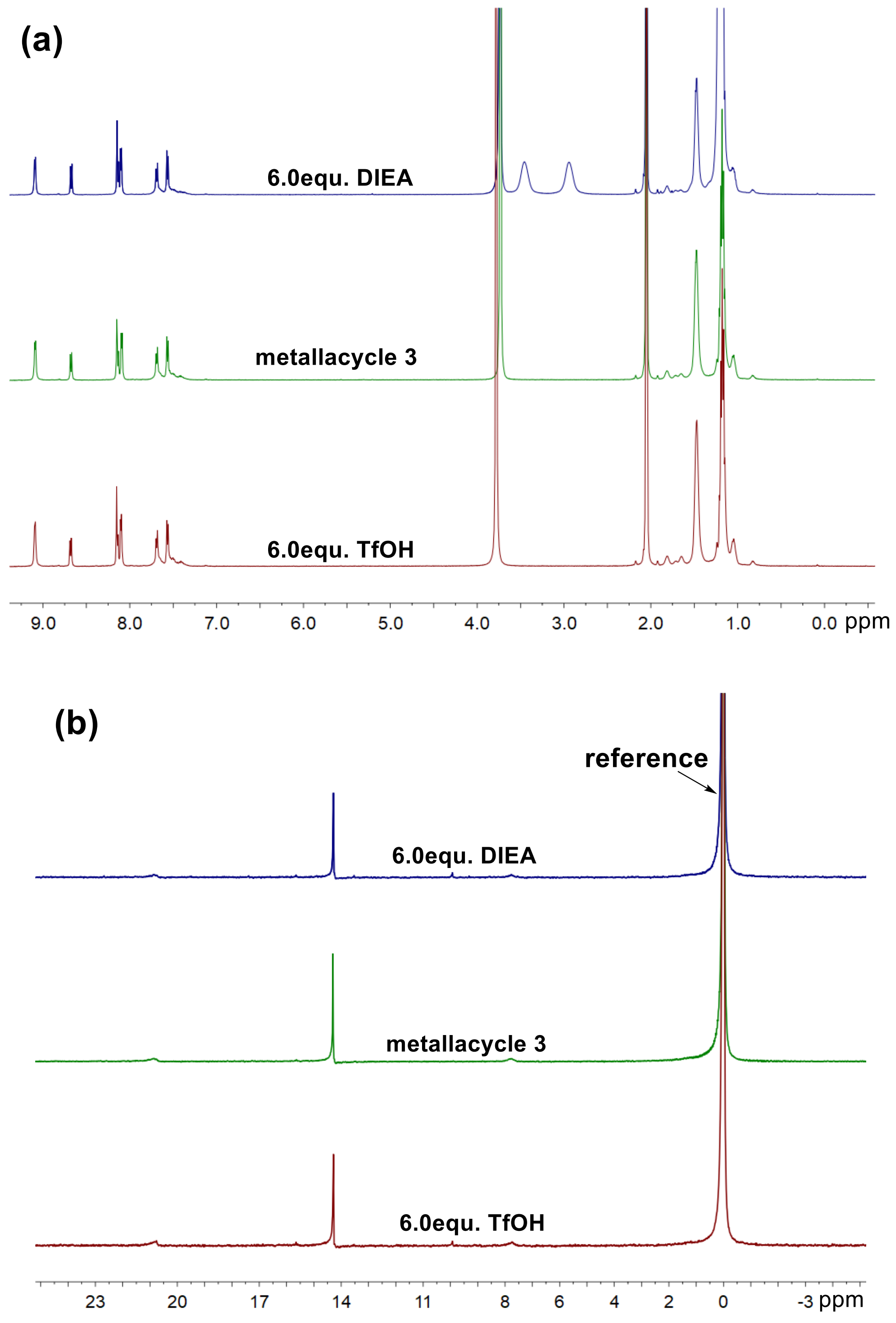

Supplementary Figure 36. (a) The stacked ^{1}H NMR spectra of metallacycle **3** before and after adding DIEA or TfOH. (b) The stacked ^{31}P NMR spectra of metallacycle **3** before and after adding DIEA or TfOH.

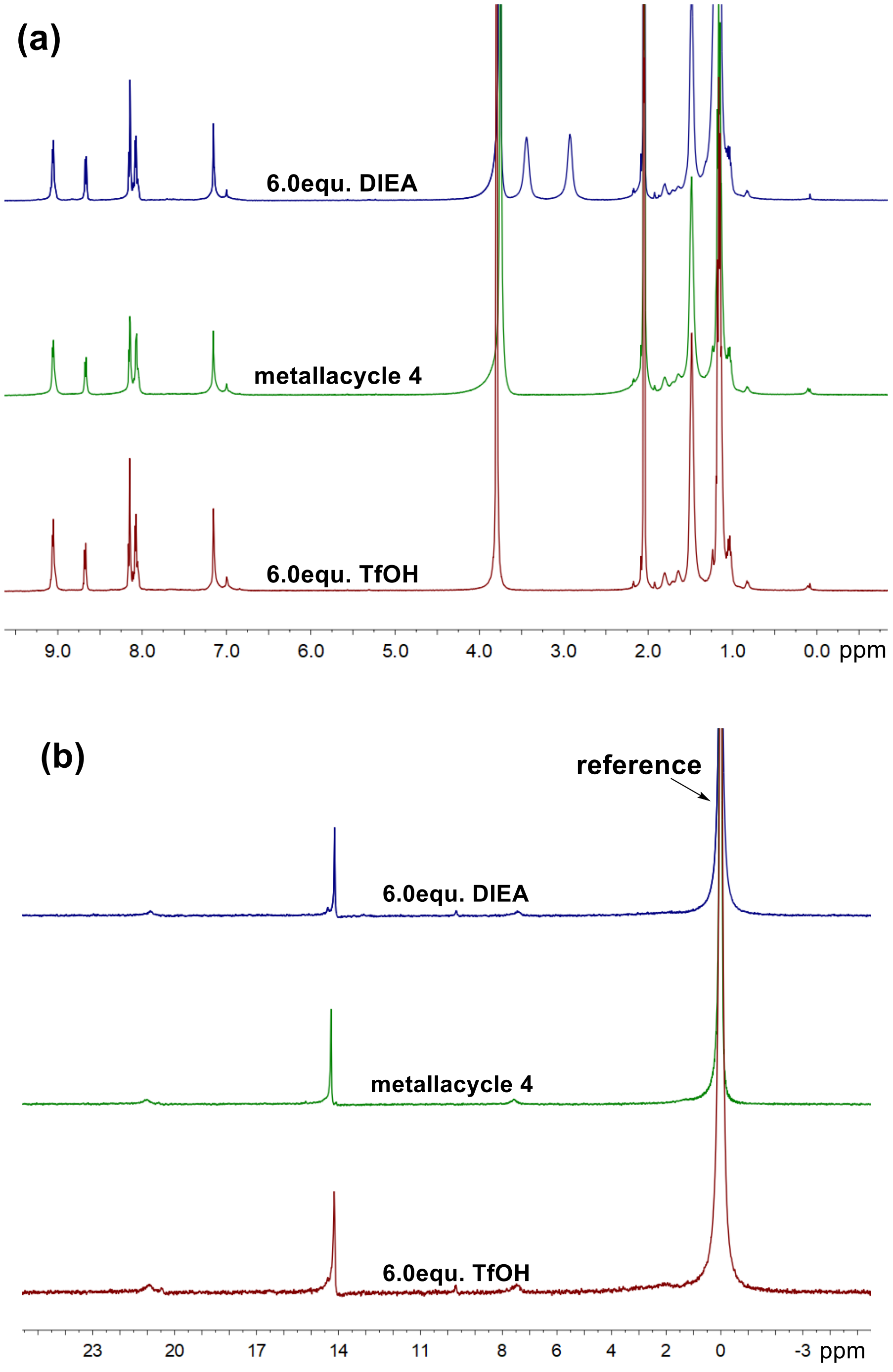

Supplementary Figure 37. (a) The stacked ^{1}H NMR spectra of metallacycle **4** before and after adding DIEA or TfOH. (b) The stacked ^{31}P NMR spectra of metallacycle **4** before and after adding DIEA or TfOH.

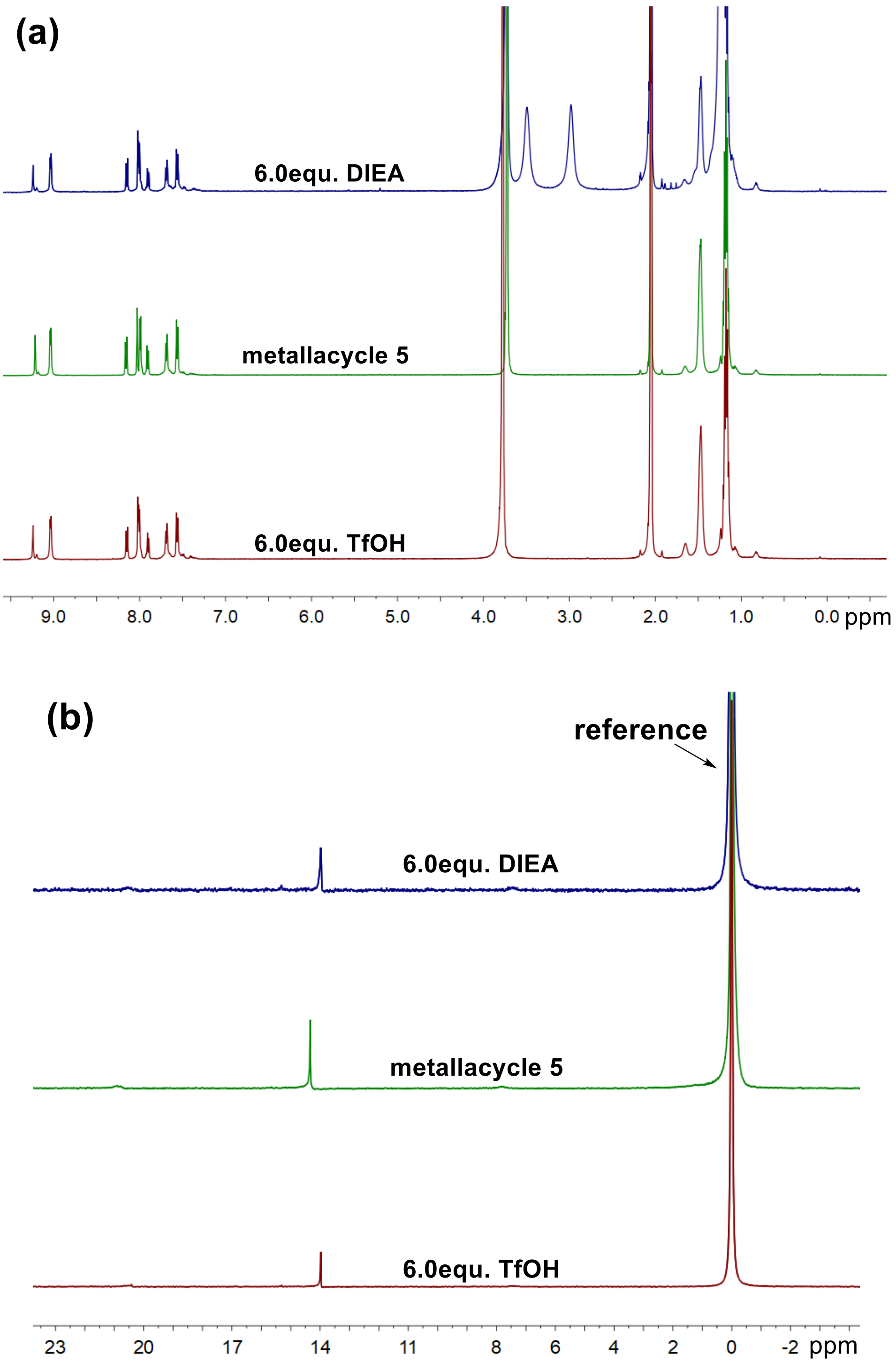

Supplementary Figure 38. (a) The stacked ^{1}H NMR spectra of metallacycle **5** before and after adding DIEA or TfOH. (b) The stacked ^{31}P NMR spectra of metallacycle **5** before and after adding DIEA or TfOH.

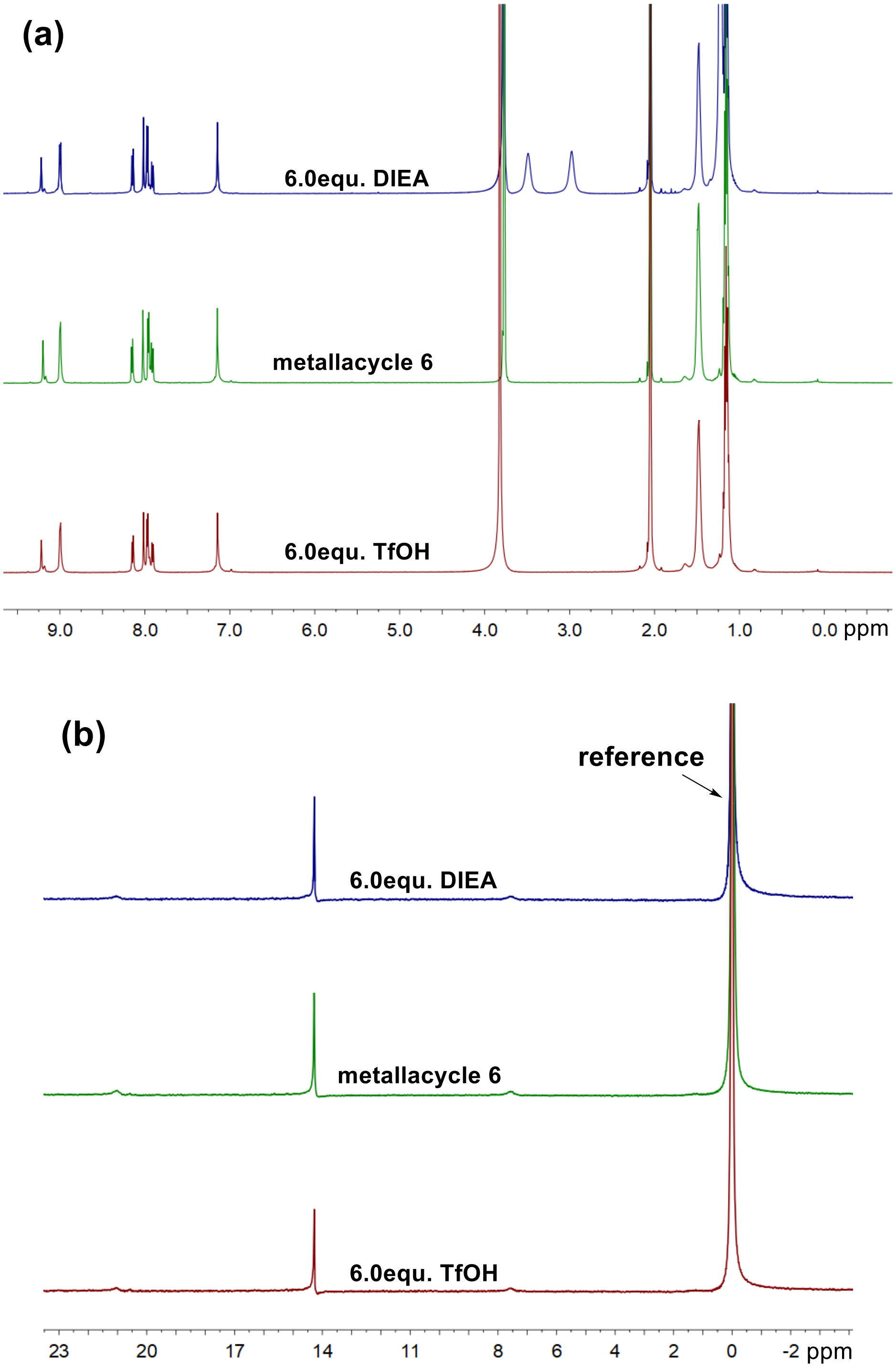

Supplementary Figure 39. (a) The stacked ^{1}H NMR spectra of metallacycle **6** before and after adding DIEA or TfOH. (b) The stacked ^{31}P NMR spectra of metallacycle **6** before and after adding DIEA or TfOH.

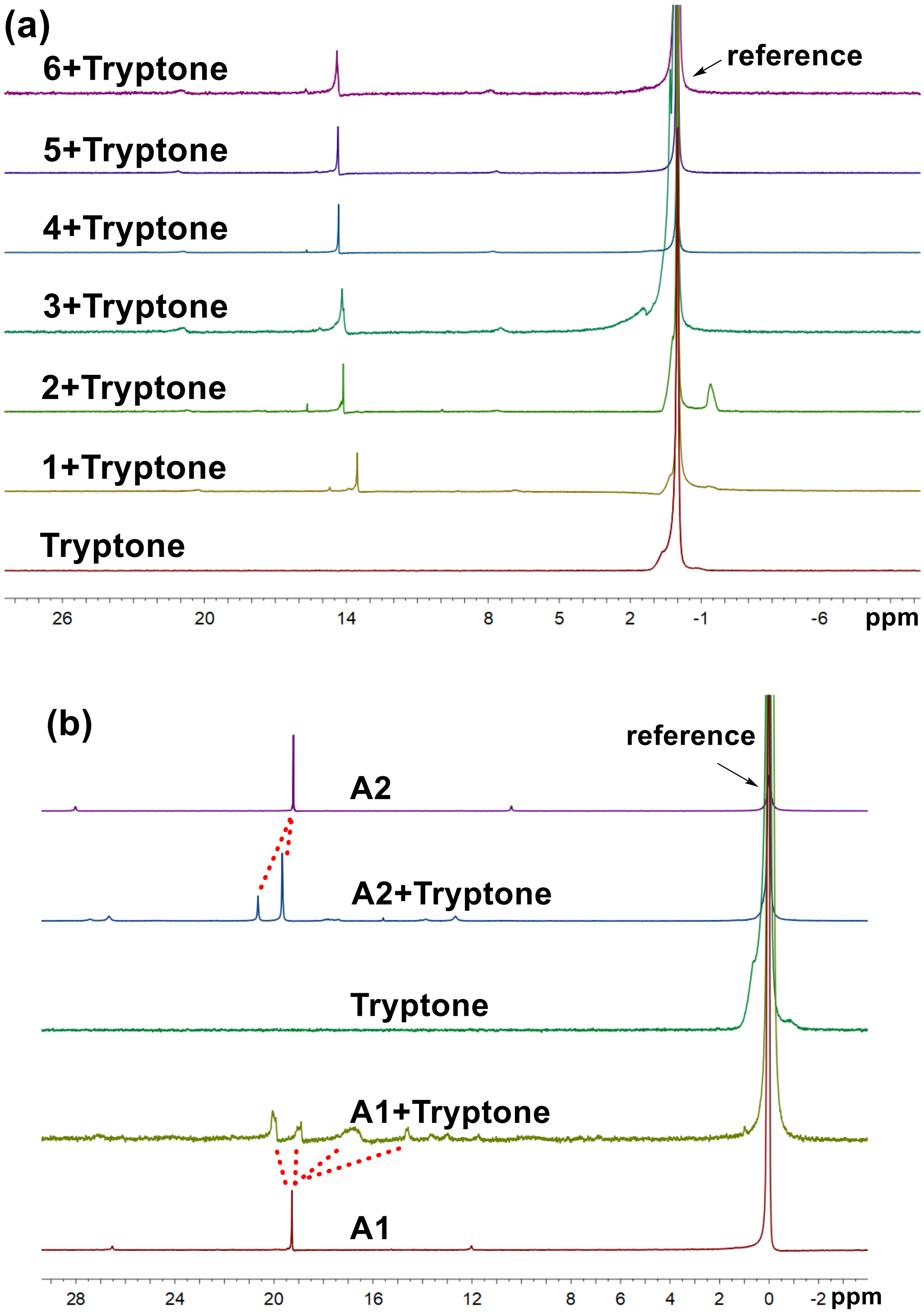

Supplementary Figure 40. (a) The stacked ^{31}P NMR spectra of all assemblies with tryptone. (b) The stacked ^{31}P NMR spectra of **A1**, **A2** with tryptone.

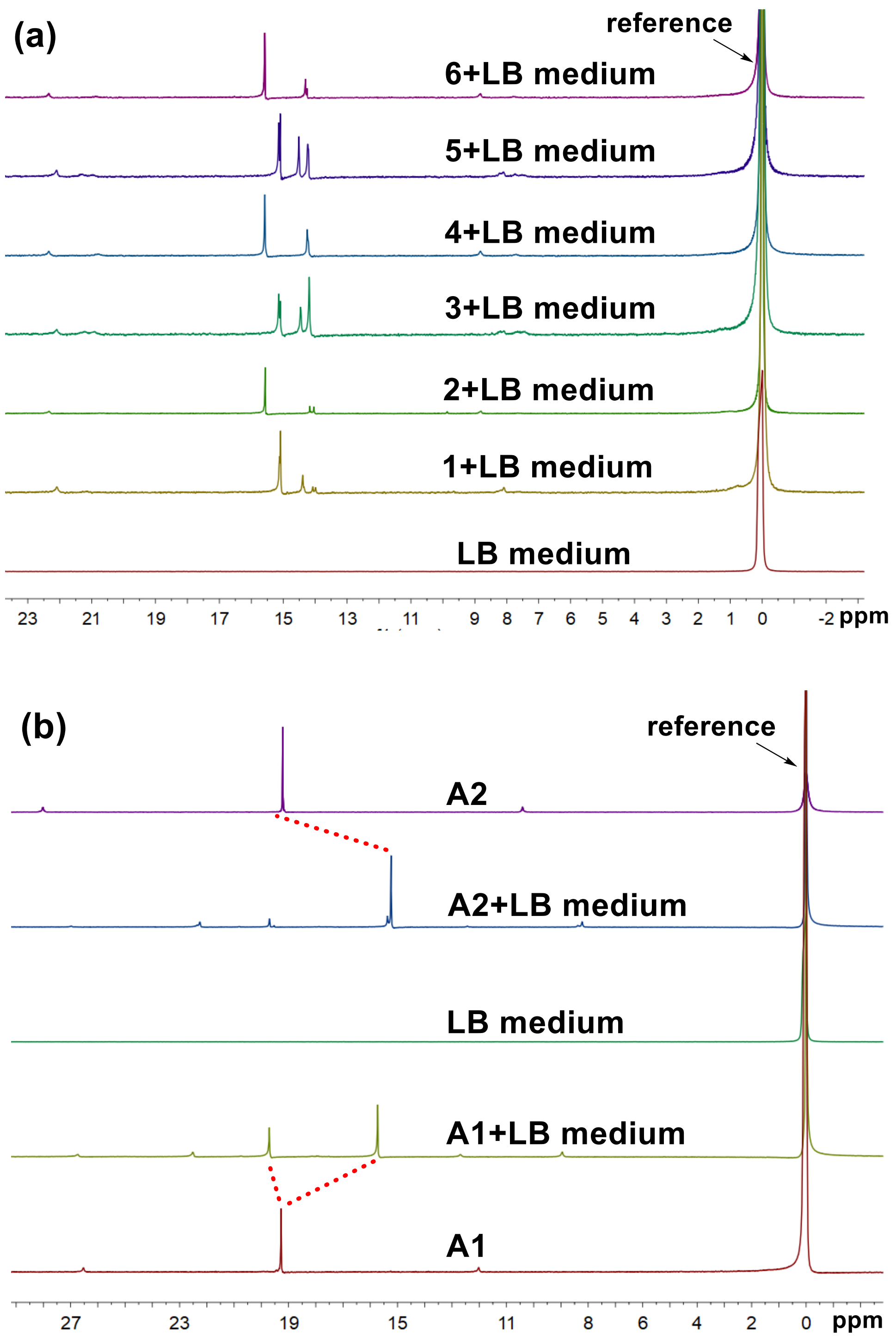

Supplementary Figure 41. (a) The stacked ^{31}P NMR spectra of all assemblies with LB medium. (b) The stacked ^{31}P NMR spectra of **A1**, **A2** with LB medium.

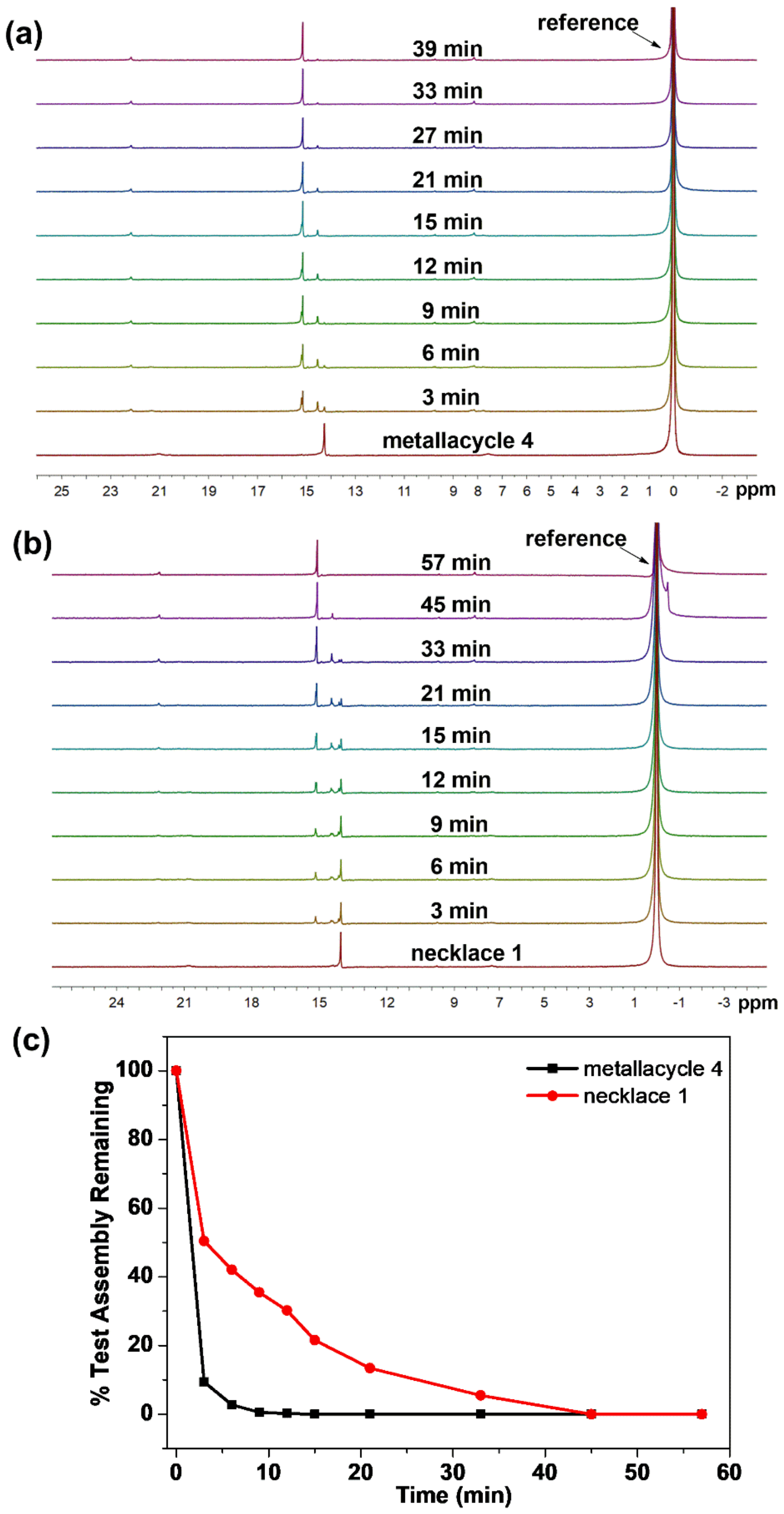

Supplementary Figure 42. Time dependent ^{31}P NMR spectra of metallacycle **4** (a) and necklace **1** (b), and their degradation curves (c).

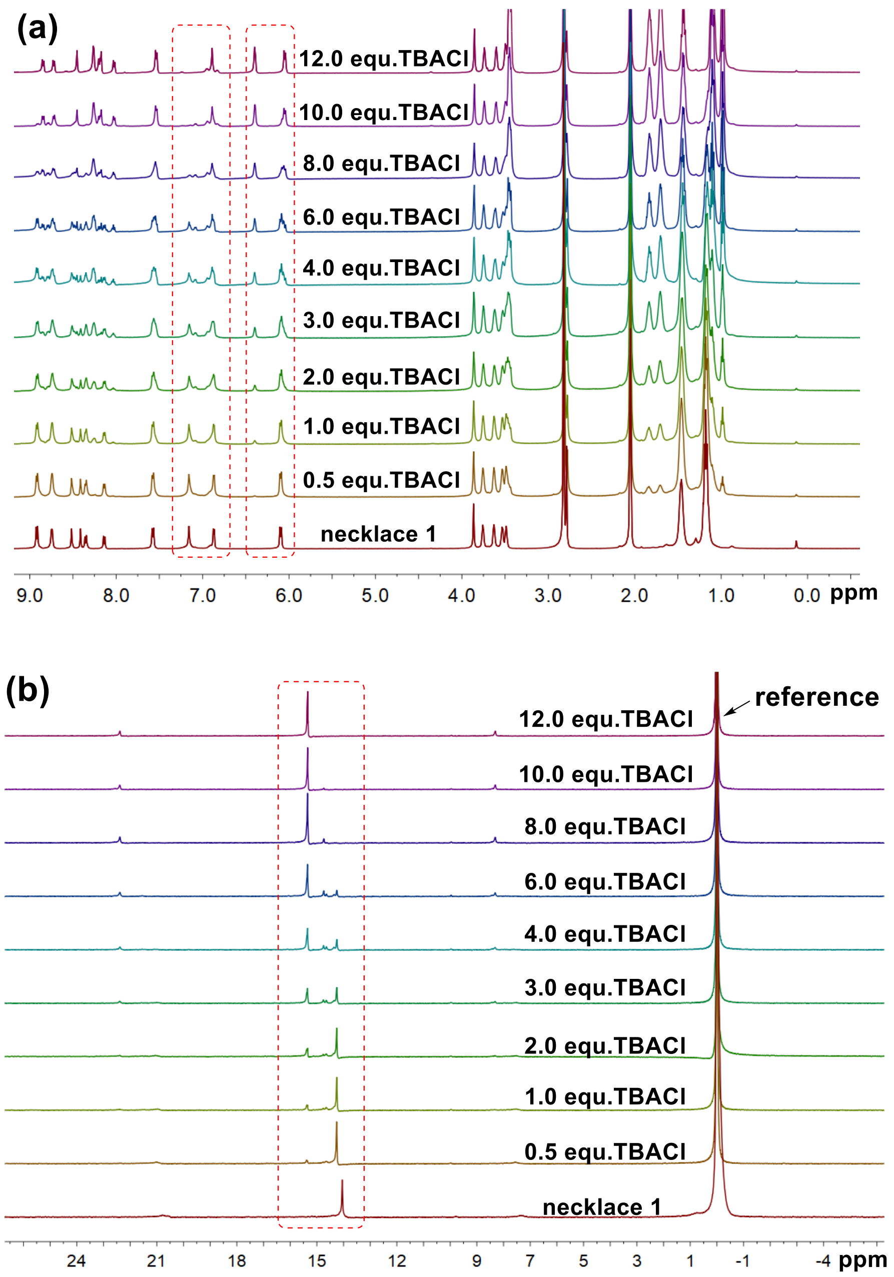

Supplementary Figure 43. Evolution of the ^{1}H NMR (a) and ^{31}P NMR (b) spectra of molecular necklace **1** upon titration with tetrabutyl ammonium chloride (TBACl).

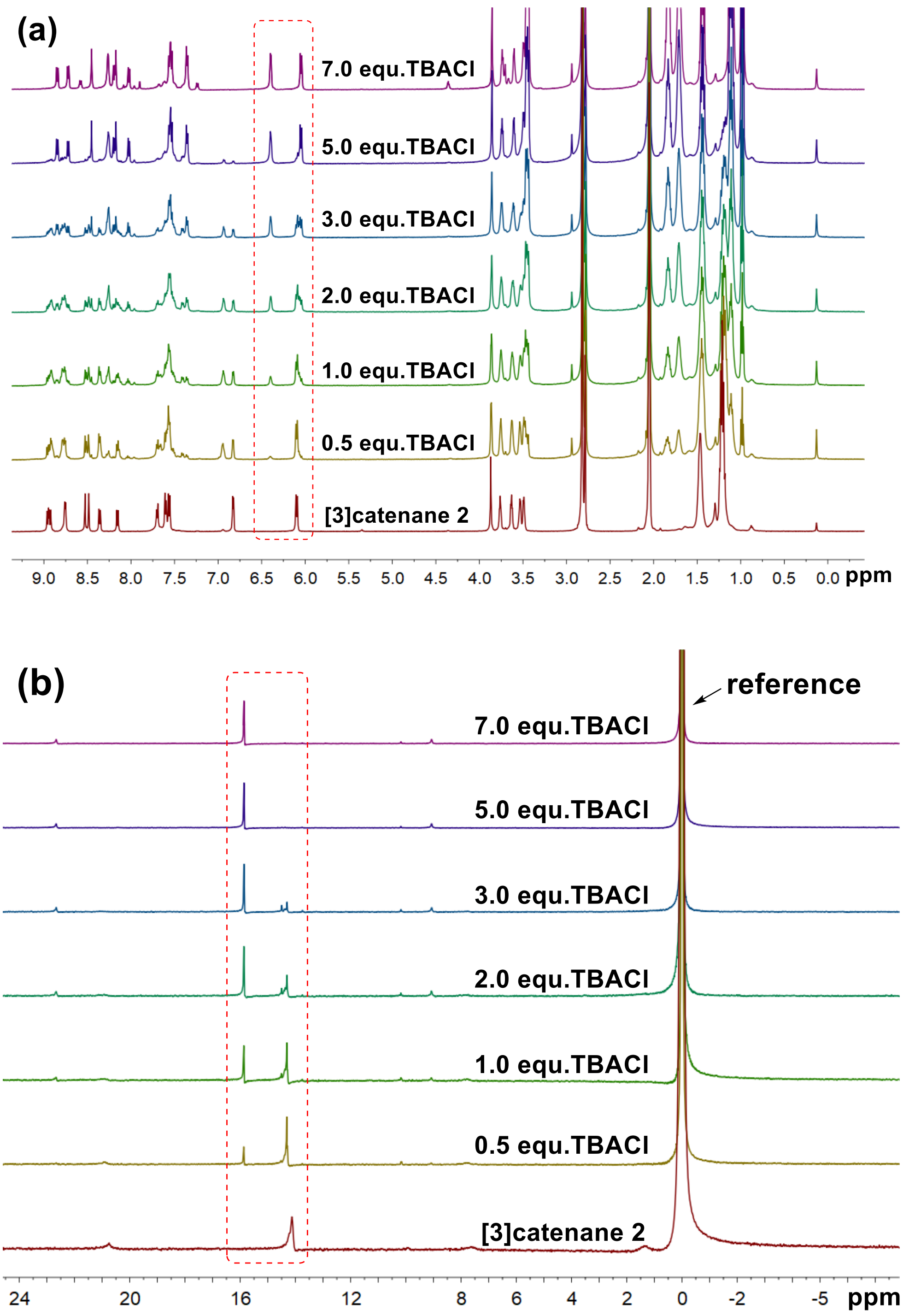

Supplementary Figure 44. Evolution of the ^{1}H NMR (a) and ^{31}P NMR (b) spectra of [3]catenane **2** upon titration with TBACl.

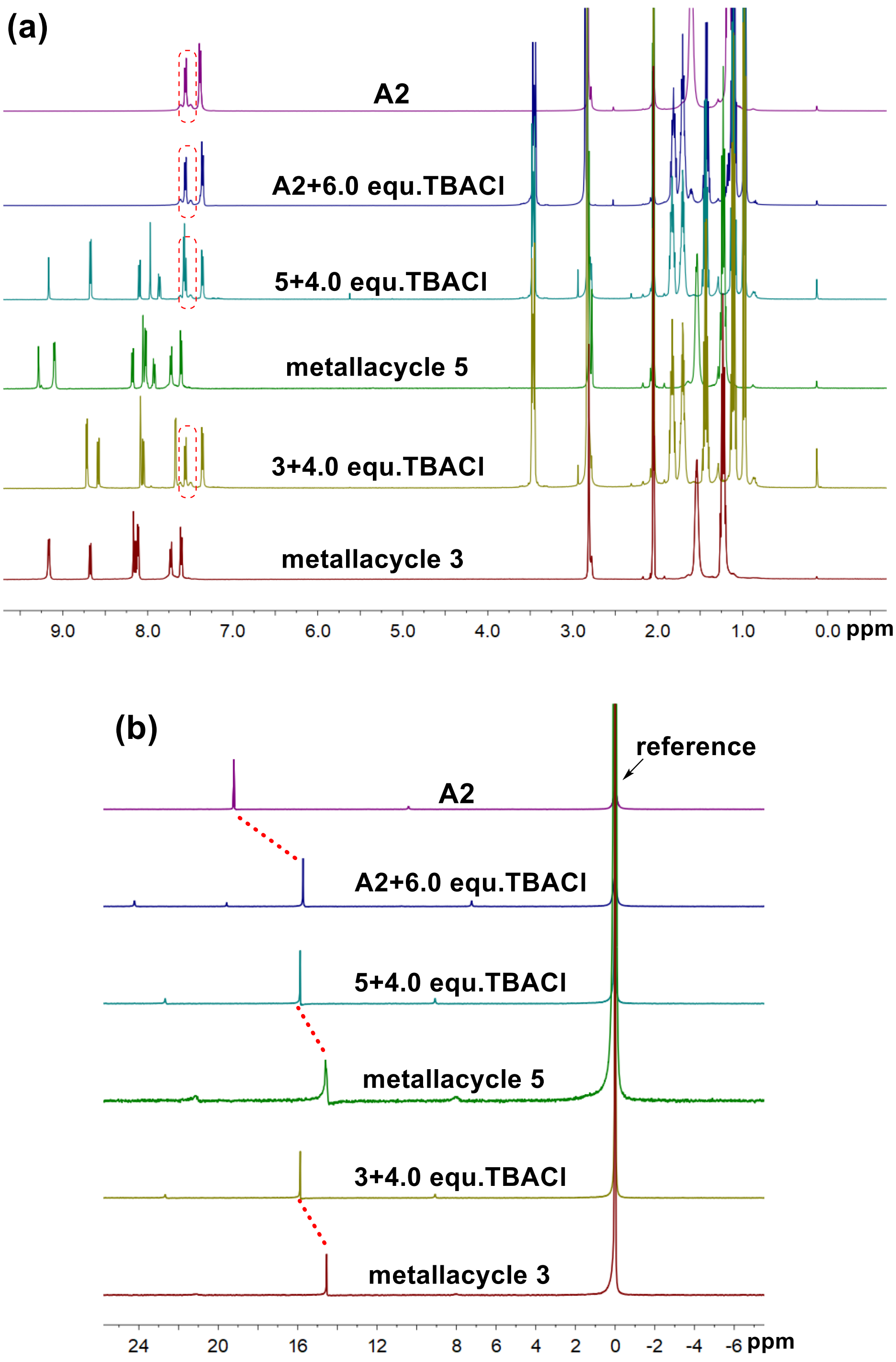

Supplementary Figure 45. (a) The stacked ^{1}H NMR spectra of metallacycle **3**, **5** and acceptor **A2** with TBACl. (b) The stacked ^{31}P NMR spectra of metallacycle **3**, **5** and acceptor **A2** with TBACl.

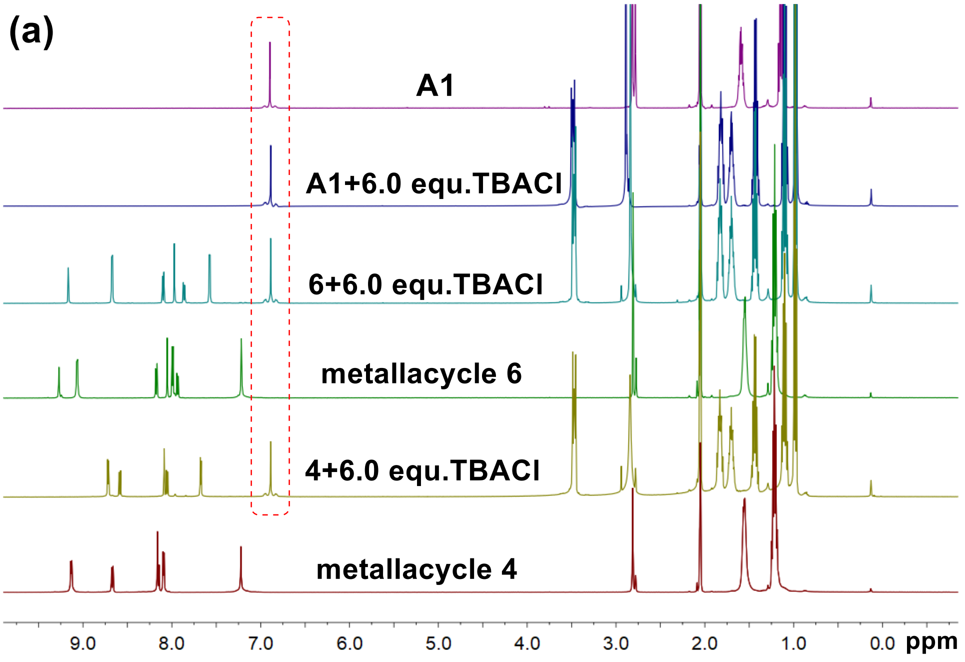

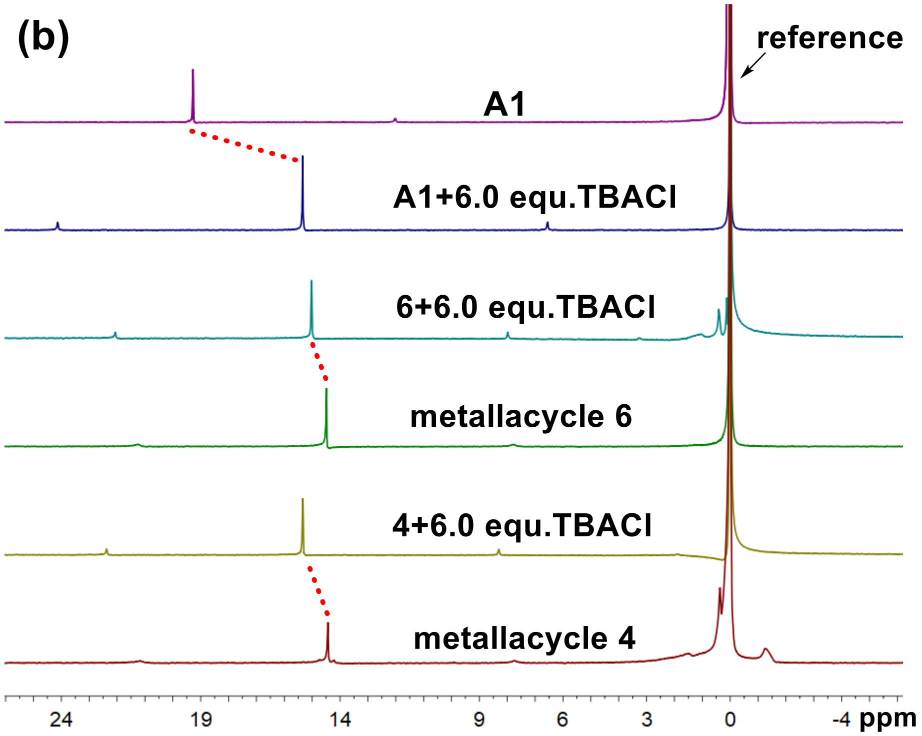

Supplementary Figure 46. (a) The stacked ^{1}H NMR spectra of metallacycle **4**, **6** and acceptor **A1** with TBACl. (b) The stacked ^{31}P NMR spectra of metallacycle **4**, **6** and acceptor **A1** with TBACl.

5

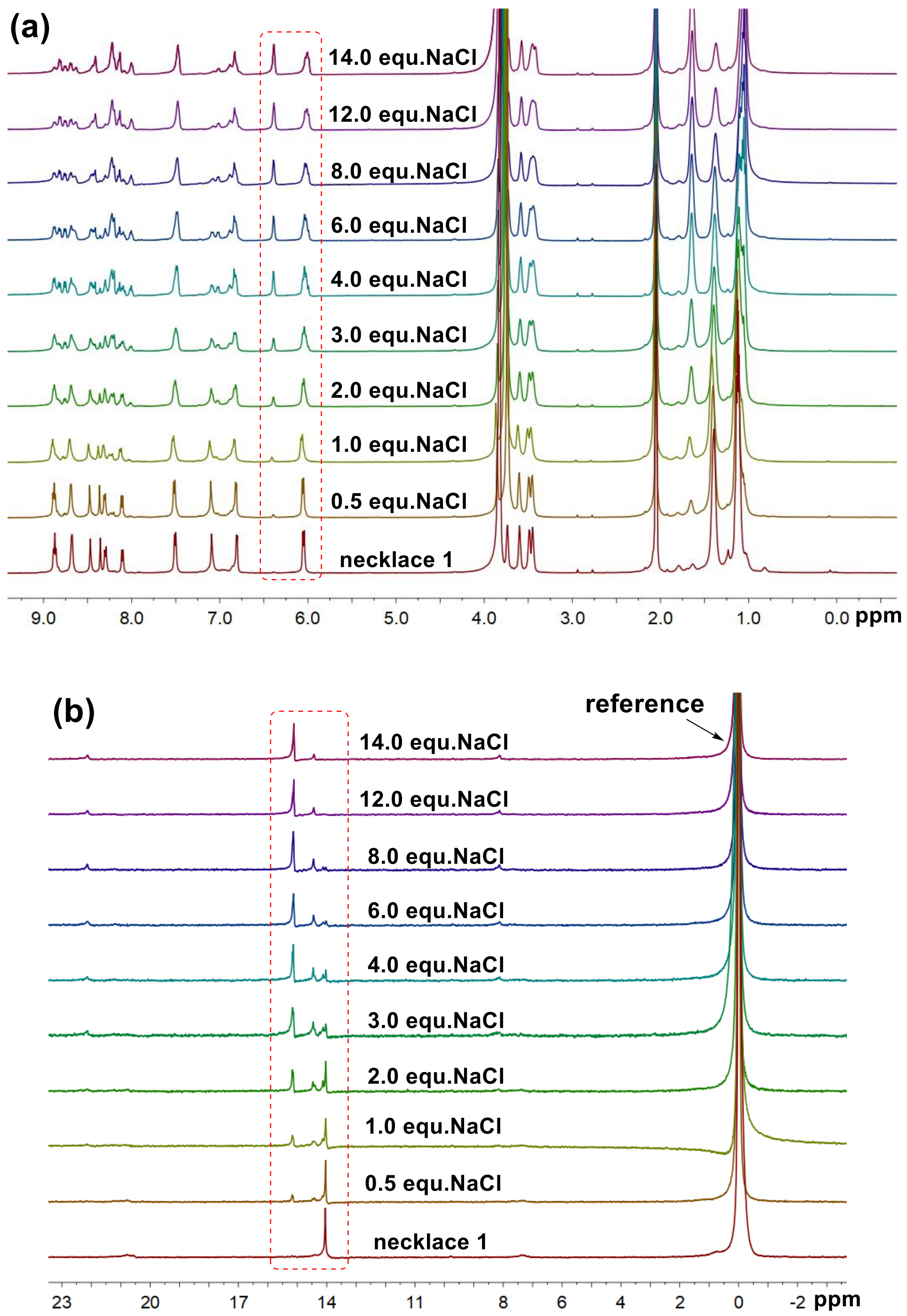

Supplementary Figure 47. Evolution of the ^{1}H NMR (a) and ^{31}P NMR (b) spectra of molecular necklace **1** upon titration with sodium chloride (NaCl).

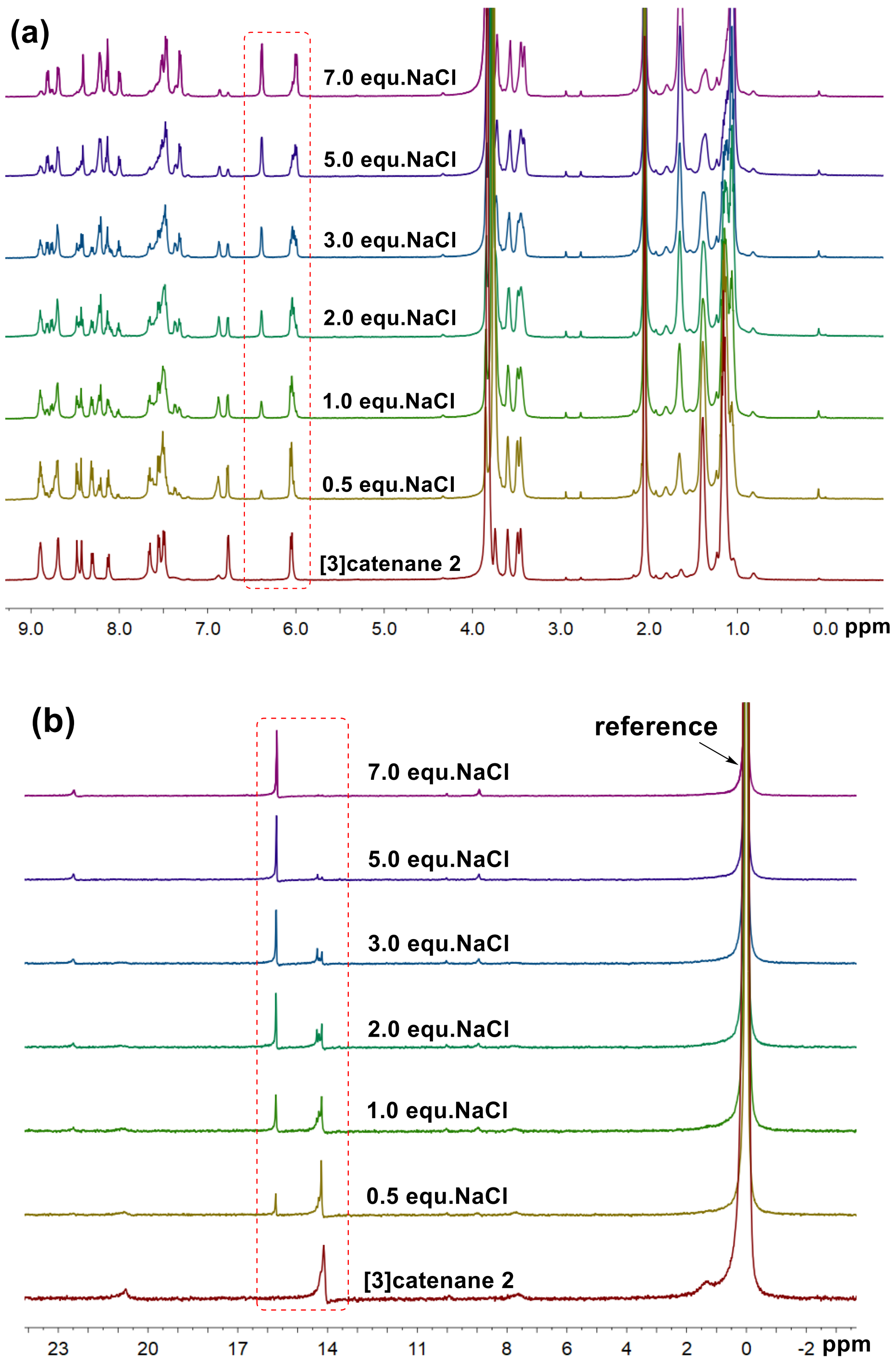

Supplementary Figure 48. Evolution of the ^{1}H NMR (a) and ^{31}P NMR (b) spectra of [3]catenane **2** upon titration with NaCl.

5

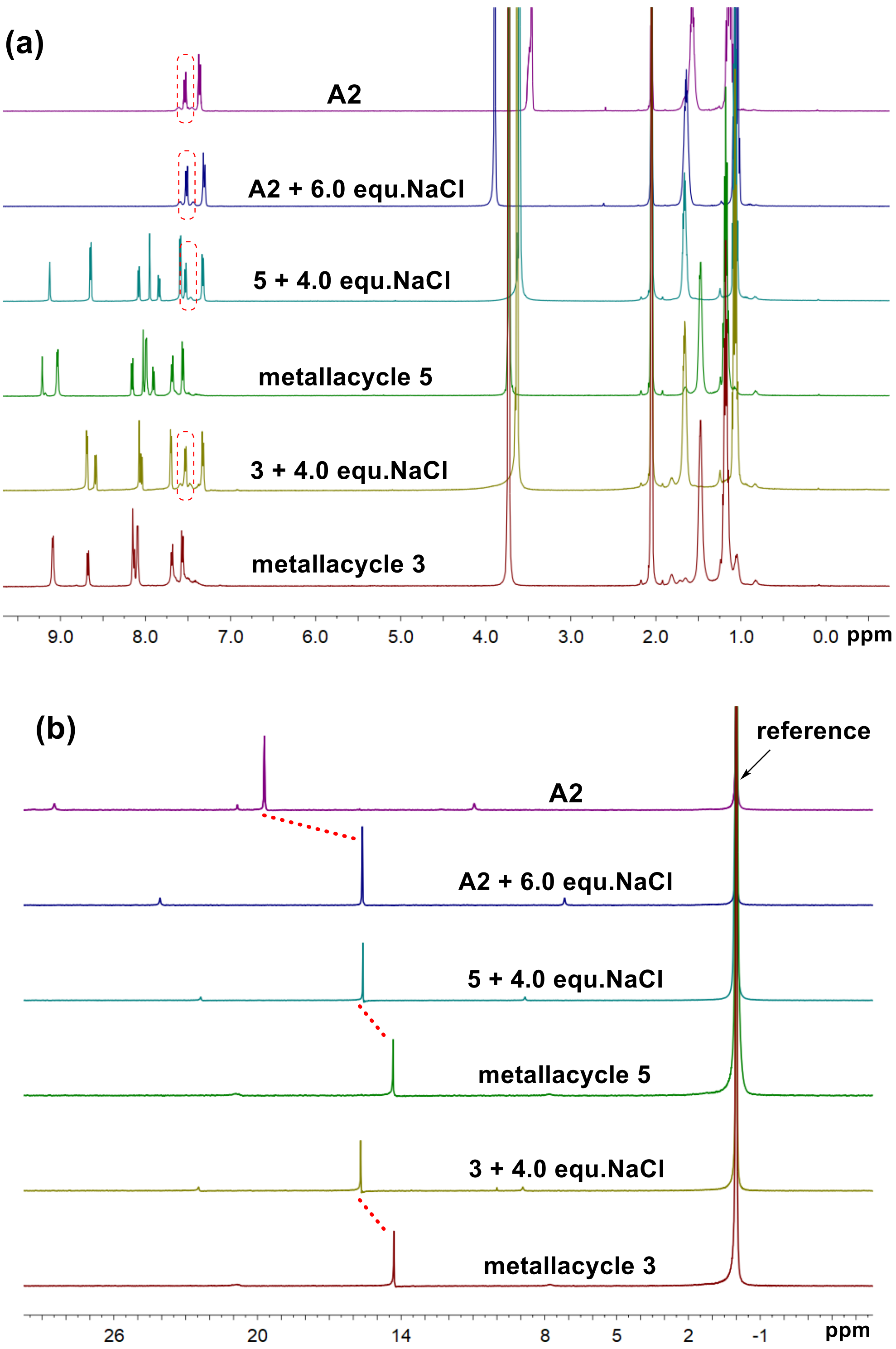

Supplementary Figure 49. (a) The stacked ^{1}H NMR spectra of metallacycle **3**, **5** and acceptor **A2** with NaCl. (b) The stacked ^{31}P NMR spectra of metallacycle **3**, **5** and acceptor **A2** with NaCl.

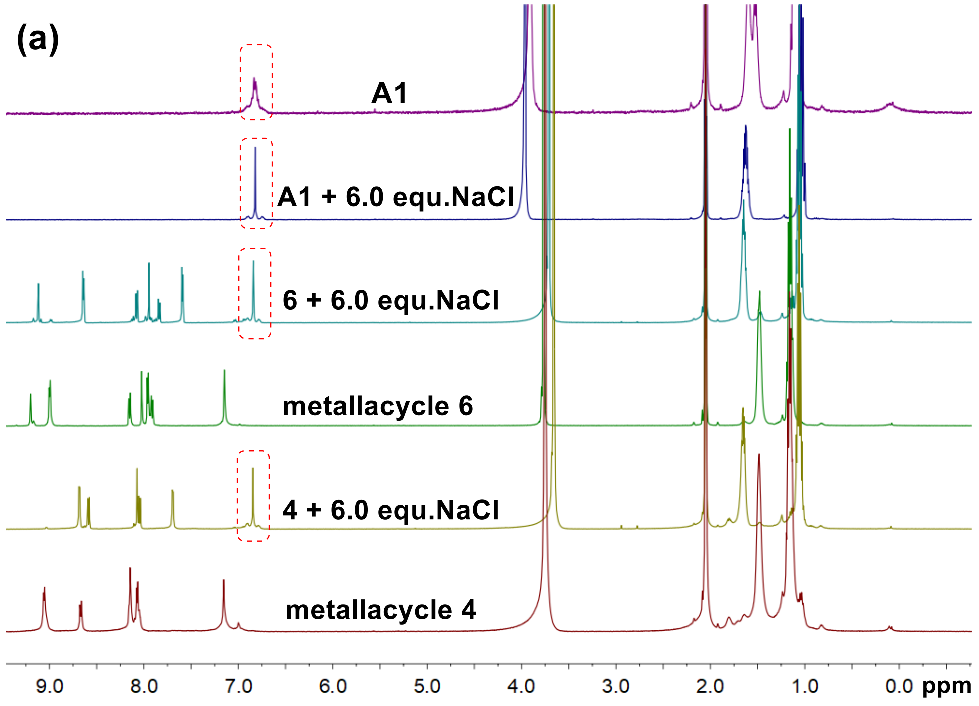

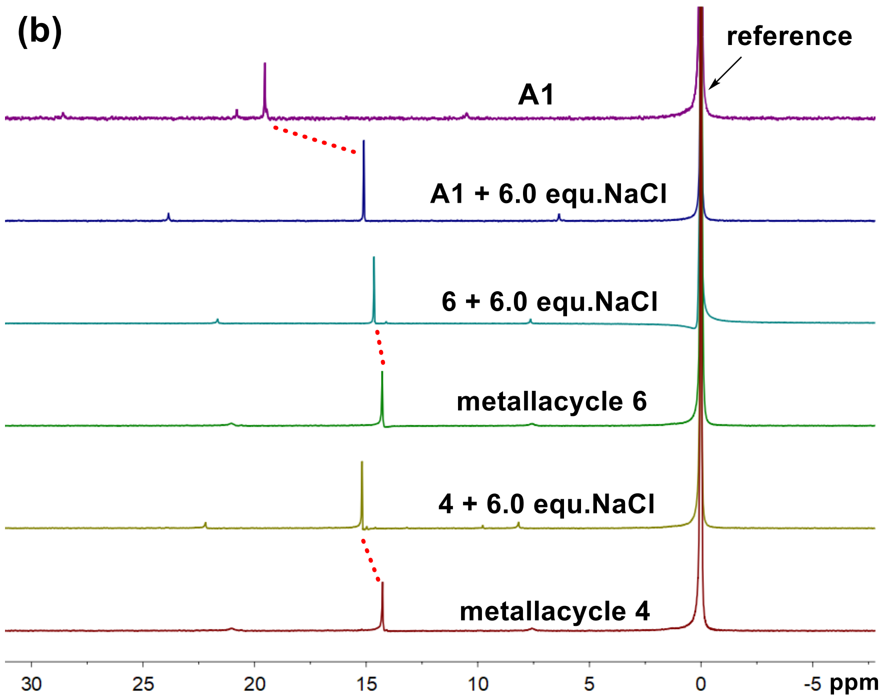

Supplementary Figure 50. (a) The stacked ^{1}H NMR spectra of metallacycle **4**, **6** and acceptor **A1** with NaCl. (b) The stacked ^{31}P NMR spectra of metallacycle **4**, **6** and acceptor **A1** with NaCl.

5
